# Characterization of Weak Protein Domain Structure by Spin-Label Distance Distributions

**DOI:** 10.3389/fmolb.2021.636599

**Published:** 2021-04-12

**Authors:** Irina Ritsch, Laura Esteban-Hofer, Elisabeth Lehmann, Leonidas Emmanouilidis, Maxim Yulikov, Frédéric H.-T. Allain, Gunnar Jeschke

**Affiliations:** ^1^ Department of Chemistry and Applied Biosciences, ETH Zürich, Zürich, Switzerland; ^2^ Department of Biology, ETH Zürich, Zürich, Switzerland

**Keywords:** low-complexity domains, RNA-binding proteins, ensemble structure, EPR spectroscopy, random coil, intrinsically disordered, liquid-liquid phase separation

## Abstract

Function of intrinsically disordered proteins may depend on deviation of their conformational ensemble from that of a random coil. Such deviation may be hard to characterize and quantify, if it is weak. We explored the potential of distance distributions between spin labels, as they can be measured by electron paramagnetic resonance techniques, for aiding such characterization. On the example of the intrinsically disordered N-terminal domain 1–267 of fused in sarcoma (FUS) we examined what such distance distributions can and cannot reveal on the random-coil reference state. On the example of the glycine-rich domain 188–320 of heterogeneous nuclear ribonucleoprotein A1 (hnRNP A1) we studied whether deviation from a random-coil ensemble can be robustly detected with 19 distance distribution restraints. We discuss limitations imposed by ill-posedness of the conversion of primary data to distance distributions and propose overlap of distance distributions as a fit criterion that can tackle this problem. For testing consistency and size sufficiency of the restraint set, we propose jack-knife resampling. At current desktop computers, our approach is expected to be viable for domains up to 150 residues and for between 10 and 50 distance distribution restraints.

## Introduction

Based on Anfinsen’s influential thermodynamic hypothesis, function of proteins was considered for a long time to depend exclusively on domains that are well represented by a single conformer with minimum free energy. Structure of such domains can be specified at atomic resolution and can be characterized by well-established techniques, such as x-ray crystallography, NMR spectroscopy, and cryo-electron microscopy. However, meanwhile it is also well-established knowledge that a substantial fraction of the genome codes for intrinsically disordered domains (IDDs) or proteins (IDPs) that are also functional, without relying for their function on three-dimensional structure defined at atomic or near-atomic resolution (Uversky, 2019). Often, IDDs and IDPs are described as structure-less, implying that they exhibit random-coil behavior similar to the one postulated for chemically denatured proteins (Fitzkee and Rose, 2004). However, this assumption is at odds with the observation that many disordered domains contain evolutionary conserved residues and that mutations in such domains can be pathogenic. This applies even to disordered domains that have not been found to fold upon binding to other proteins or nucleic acids, suggesting that conformation space of proteins cannot be described by a dichotomy of atomic-resolution structure and complete absence of structure. Instead, there exists a continuum between order and disorder (Uversky et al., 2005; Habchi et al., 2014). IDDs are ubiquitous in nucleic-acid binding proteins, as has been demonstrated early for transcription factors (Liu et al., 2006). More recently, formation of membrane-less organelles by RNA-binding proteins has been associated with the presence of IDDs (Brangwynne et al., 2015; Uversky, 2017). Despite impressive development of characterization techniques for IDDs over the past two decades, quantification of weak deviation from random-coil behavior has remained a complicated problem. In a dynamic picture, such weak order corresponds to correlated motion of residues or groups of residues with large sequence separation that would not be expected in a random coil (Kurzbach et al., 2016). In an ensemble-average picture, this correlated motion is expected to cause heterogeneity of segmental radius of gyration across the peptide sequence. In other words, sections of the chain will be more or less compact, depending on where in the sequence they are situated.

While the radius of gyration is easy to measure for the whole chain, such measurements are not feasible for sections of the chain. However, it is well known from polymer physics that the radius of gyration and the root mean square (RMS) end-to-end distance both scale by an exponential law with respect to the number of residues, with the scaling exponent coinciding for ideal chains. The end-to-end distance of chain sections is accessible by distance measurements between spin labels using pulsed dipolar spectroscopy (PDS) techniques, in particular by the double electron resonance (DEER) experiment. Such measurements are well established for proteins (Schiemann and Prisner, 2007; Jeschke, 2012). The primary data that they provide can be processed into distance distributions (Jeschke et al., 2002; Bowman et al., 2004; Chiang et al., 2005), which can in turn be predicted from polymer physics models (Zheng et al., 2018) and from ensemble models of a domain (Jeschke, 2016). Such characterization by distance distribution measurements has been applied to IDPs in the past (Drescher, 2012; Geist et al., 2013). Here we develop a general approach for using distance distribution data in ensemble modeling and for analyzing such ensembles in terms of weak order.

The first approaches for experimentally informed ensemble modeling of intrinsically disordered proteins utilized small-angle x-ray scattering curves (Bernado et al., 2007) or ensemble mean values of NMR parameters, such as various chemical shifts, ^3^J_HNHa_ couplings, residual dipolar couplings, paramagnetic relaxation enhancements, and nuclear Overhauser enhancements (Marsh and Forman-Kay, 2012). The maximum entropy principle allows for combining molecular dynamics (MD) simulations with any type of constraints or restraints for which a forward model exists that can compute the measured quantity from atomic coordinates of a conformer (Cesari et al., 2018). Alternatively, ensembles resulting from unbiased MD simulations or other sampling techniques can be reweighted based on the maximum entropy principle (Köfinger et al., 2019). A somewhat similar approach, which has been discussed for DEER data, estimates the maximum occurrence of a conformer in an ensemble that is consistent with experimental restraints (Gigli et al., 2018). Principles of modeling proteins ensembles have been recently reviewed (Bonomi et al., 2017).

Our approach is based on the idea that knowledge of the probability distribution of experimental restraints, as is the case with DEER distance distributions, provides us with additional information compared to ensemble mean values. Hence we can apply our restraints as “distribution restraints”. In a first modeling step, distribution restraints can guide sampling, as they allow for testing to what extent a single conformer is consistent with experimental data (Jeschke, 2016). In a second step, they can be used, together with other types of restraints, in ensemble reweighting (Jeschke, 2021). For application work, such integration with other types of data is desired and may often be necessary in order to sufficiently restrain the model and test its reliability. In the present study, we develop an approach that provides ensemble models from only distance distribution data and tests their robustness.

This development faces three obstacles. As a first obstacle, the reference state of complete disorder is assumed, but not proved to correspond to a Flory self-avoiding walk random coil. We need to test whether this approximation is sufficiently good in the range where label-to-label distance distribution measurements can be performed. To that end, we analyze data from a recent study on liquid-liquid phase separation (LLPS) of the intrinsically disordered N-terminal domain (NTD) of fused in sarcoma (FUS), where sections 10–29 and 105-128 exhibited featureless, Gaussian-like distance distributions (Emmanouilidis et al., 2021). Specifically, we address the question whether PDS EPR can distinguish between Gaussian-like distributions and self-avoiding walk distributions with variable scaling exponent ν (SAW-ν distributions) within the segment-length range where its distance distribution fidelity is best. This question is important for specifying restraints in modeling. As a second obstacle, conversion of primary data to distance distributions is an ill-posed problem (Jeschke et al., 2002; Chiang et al., 2005; Gigli et al., 2018), which may be particularly difficult to solve for very broad distributions that extend to rather long distances (Jeschke et al., 2004). We address this problem by systematic analysis of 19 distance distributions obtained on heterogeneous nuclear ribonucleoprotein A1 (hnRNP A1), a human protein predominantly active in mRNA regulation (Jean-Philippe et al., 2013). HnRNP A1 consists of a glycine-rich domain (188–320), which is an intrinsically disordered low-complexity domain (LCD) with a similar amino acid composition as the NTD of FUS, tethered to two folded RNA recognition motifs (RRMs). We analyze DEER data obtained with one or both label sites in the LCD of hnRNP A1 with different approaches and check how this influences ensemble modeling. Apart from addressing data analysis, hnRNP A1 also provides a reference case for analyzing weak structure in flexible domains tethered to folded domains with known structure. This is achieved by including the previously reported solution structure of the RRMs (Barraud and Allain, 2013) in the modeling. As a third obstacle, it is difficult to estimate how many distance distributions are required for sufficiently restraining an ensemble model in the regime of weak order. In order to answer this question, we perform jack-knife resampling, where systematically one of the 19 restraints for hnRNP A1 is left out and the corresponding distance distribution is predicted by an ensemble model fitted to the other 18 restraints. Finally, we discuss limitations of our approach as well as possible extensions.

## Materials and Methods

### Sample Preparation for hnRNP A1

A construct of His_6_-tagged wild-type hnRNPA1 (P09651, isoform A1-A), was already available in the Allain group at ETH Zurich, and a two-step affinity chromatography protocol was adapted from (Barraud and Allain, 2013) to purify all mutants. Further experimental details can be found in (Ritsch, 2019). In summary, point mutations of the partially buried native Cys in the folded domains to non-reactive residues (C43S, C175A), and to introduce pair-wise engineered Cys spin labeling sites were introduced by sequential application of site-directed mutagenesis with PCR primers with the desired point mutation. Spin labeling was performed with MTSL (2,2,5,5-tetramethyl-3-pyrroline-3-methylmethanethiosulfonate) in 50 mM sodium phosphate buffer, pH 6.5, 100 mM L-arginine, and 100 mM L-glutamate at approximately 10 times molar excess and 10 μM hnRNP A1 concentration. Excess spin label was removed on a PD10 desalting column (GE Healthcare), and the labeled protein was concentrated in 10 kDa MWCO centricons (Amicon Ultra-4 Centrifugal Filter Units, Merck and Cie). DEER samples were prepared by mixing with d8-glycerol (1:1 ratio, v:v), and transferring ∼35 μL solution at approximately 25 μM hnRNP A1 concentration to 3 mm outer diameter quartz capillaries and flash-freezing by immersion into liquid nitrogen.

### DEER Measurements on hnRNP A1

The DEER measurements were performed at a home-built high power Q-band spectrometer (≈34 GHz) controlled by a Bruker Elexsys E580 bridge in a home-built TE001-type resonator at 50 K. The 4-pulse DEER pulse sequence was used, with pulse lengths of *t*(π) = *t* (π/2) = 16 ns. The pump pulse length was either 16 or 12 ns (on the maximum of the nitroxide spectrum), and a pump/detection frequency separation of 100 MHz was used. The first refocusing delays was *t*_1_ = 400 ns, and the second refocusing delay was set individually for each sample (as long as possible to be able to still detect a reasonably strong echo). The time-step was either 12 or 8 ns.

### Distance Distribution Analysis

The sample preparation and DEER measurements of FUS are described in detail in (Emmanouilidis et al., 2021). Distance distribution analysis for FUS was performed using DeerLab 0.9.0 (downloadable at jeschkelab.github.io/DeerLab/). Briefly, we used a multi-pathway kernel K(t,r) of the form

$$K\left(t,r\right)=\left[{\text{Λ}}_{0}+\lambda_{1}K_{0}\left(t,r\right)+\;\lambda_{2}K_{0}\left(t-T_{0,2},r\right)\right]e^{-k\left(\lambda_{1}\left|t\right|+\lambda_{2}\left|t-\;T_{0,2}\right|\right)}$$

where $K_{0}$ is the elementary dipolar kernel, $\Lambda_{0}$ accounts for the contribution of unmodulated dipolar pathways, *λ*_*1*_ and *λ*_*2*_ describe the amplitudes of the modulated dipolar pathways, *T*_*0,2*_ is the refocusing time of the additional modulated dipolar pathway, and *k* is the background decay rate. While *K*_0_ is fixed, Λ_0_, λ_1_, λ_2_, and *k* are fit parameters. We assumed an exponential background function. The distance distribution P(r) was fitted by Tikhonov regularization (using either the Bayesian information criterion or the residual method for regularization parameter selection), by fitting a single Gaussian distribution by varying mean distance and full width at half maximum Γ, or by fitting the SAW-ν model by varying RMS end-to-end distance and scaling exponent ν. Standard deviations σ_*r*_ were computed as ${\text{σ}}_{r}=\text{Γ}/\sqrt{8ln2}$. The dispersed phase data were taken not from the biphasic sample, but from monophasic dispersed FUS 1–267 sample in 0.6 M urea that had been prepared for obtaining initial values and lower and upper bounds for fitting biphasic data, as shown in (Emmanouilidis et al., 2021). Distance distribution analysis for hnRNP A1 was performed by single-Gaussian fitting in DeerAnalysis 2019 using default settings, by single-Gaussian and Tikhonov analysis in DeerLab 0.8b, and by DeerNet from Spinach version 2.5.5446. For Tikhonov analysis, regularization parameters were either selected by the AIC criterion or fixed at α = 5. In DeerAnalysis and DeerLab, a monoexponential background function was assumed corresponding to a homogeneous distribution of remote spin labels in three-dimensional space. DeerNet is trained with stretched exponential background functions (Worswick et al., 2018), thus also allowing for fractal dimensionality lower than three of the spatial distribution of remote labels. Results of single-Gaussian analysis with LongDistances^1^ were provided by Christian Altenbach and results of multi-Gaussian analysis by DD (Stein et al., 2015) were provided by Eric Hustedt.

### Generation of Raw Ensembles

Raw ensembles were generated in MMM using the Domain Ensemble Modeller described in (Jeschke, 2016). Briefly, in unrestrained mode the Domain Ensemble Modeller generates peptide backbone models that conform to residue-specific Ramachandran statistics for backbone torsions ϕ and ψ as provided by (Hovmoller, 2002). In restrained mode, each distance distribution restraint is tested in a given conformer as soon as backbone coordinates for both sites are available. The mean spin label position at the newly generated site is predicted from backbone coordinates, the label-to-label distance is computed, and a probability is estimated for this distance to be consistent with a Gaussian restraint. If the product of these probabilities for all evaluated restraints drops below a certain threshold, the conformer is discarded and generation of a new conformer is started. As such rejection of conformers typically occurs well before they are completely generated, the approach improves sampling of the part of conformational space that is consistent with the restraints.

Restraints were set as ⟨*r*⟩, √2σ_*r*_ and an acceptance threshold corresponding to probability 0.75 was used. For FUS, a fixed number of conformers was generated in a single run. For FUS 1–267, this number was 90 and in all other cases it was 2,500. For unrestrained FUS 1–100 and 67–166, two runs with 2,500 conformers each were performed. For hnRNP A1, PDB structure 2LYV of UP1 (Barraud and Allain, 2013) was used for the RRMs. Coordinates of only residues 1–187 were kept. In all runs, LCD models were generated for all 20 models in the NMR structure, expending 1 h computation time on eight processor cores per NMR model. This generated about 400 conformers. For the unrestrained ensemble, 48 min were expended per NMR model, leading to 2,146 conformers.

### Ensemble Fitting

Ensemble fitting was performed with the *module_ensemble_fit* function of MMMx (github.com/gjeschke/MMMx, commit 55a7fef). Distance distribution files were provided in a four column format with the first column being the distance axis (units of nanometers), the second column being the distance distribution, and the third and fourth column the lower and upper bound of the uncertainty band (used only for plotting). The only change with respect to the corresponding module in MMM, as described in (Jeschke, 2021), is adaptive block size in iterative fitting. If less than 10% of the specified block size are available for the next iteration, block size is increased by 50%. If the number of retained conformers later drops, block size is reduced to the originally specified value, which was 100 in our computations.

## Results

### The Random-Coil Reference State

#### Probing the Reference State by DEER Distance Distributions

Before we can address the problem of weak structure, we need to establish what can be inferred by DEER distance distributions on an unstructured protein. In particular, we are interested in the question what level of detail can be realistically interpreted in an ensemble model informed by such distributions. We analyze this on the example of the NTD of FUS, where we used protein constructs that do not contain any well-structured domains and are thus well suited as a fully disordered reference system.

We define structure as deviation from the maximum-entropy state of a protein domain under given solvent conditions. From a polymer physics view, this maximum-entropy state corresponds to a random coil. Since protein domains are not homopolymers, some deviation from simple polymer physics models is expected. For instance, Ramachandran angle preferences are residue-specific even in loop regions of proteins (Hovmoller et al., 2002), suggesting that the maximum-entropy state of a domain depends on sequence. Therefore, the random-coil reference state itself must be characterized first before conclusions can be drawn on weak structure.

It has been demonstrated as early as 2004 that randomization of backbone torsion angles of structured proteins leads to ensembles whose RMS end-to-end distances *R* and radii of gyration *R*_G_ conform to expectations for random coils, with a scaling law $R\propto N^{\nu }$ where *N* is the number of residues and ν≈0.6 for chemically denatured proteins (Fitzkee and Rose, 2004). These conditions correspond to a Flory random coil in a good solvent where it maximally expands (ν=3/5). A single-molecule Förster resonance excitation transfer (FRET) study found ν≈0.62 for denatured and IDPs, whereas foldable sequences in water exhibited ν≈0.48, close to the condition of a Θ-solvent (ν=1/2) that offsets monomer-monomer interactions (Hofmann et al., 2012). Accordingly, the distribution *P*(*r*) of end-to-end distances in IDDs is expected to conform to a self-avoiding random walk. Dependence of chain dimension on solvent quality is then quantified by the scaling exponent ν. An expression for *P*(*r*) as a function of *R* and ν has been derived (SAW-ν model) and compared to molecular dynamics (MD) simulations (Zheng et al., 2018). The distribution is skewed with a steeper flank toward short distances than toward long distances.

**FIGURE 1:**
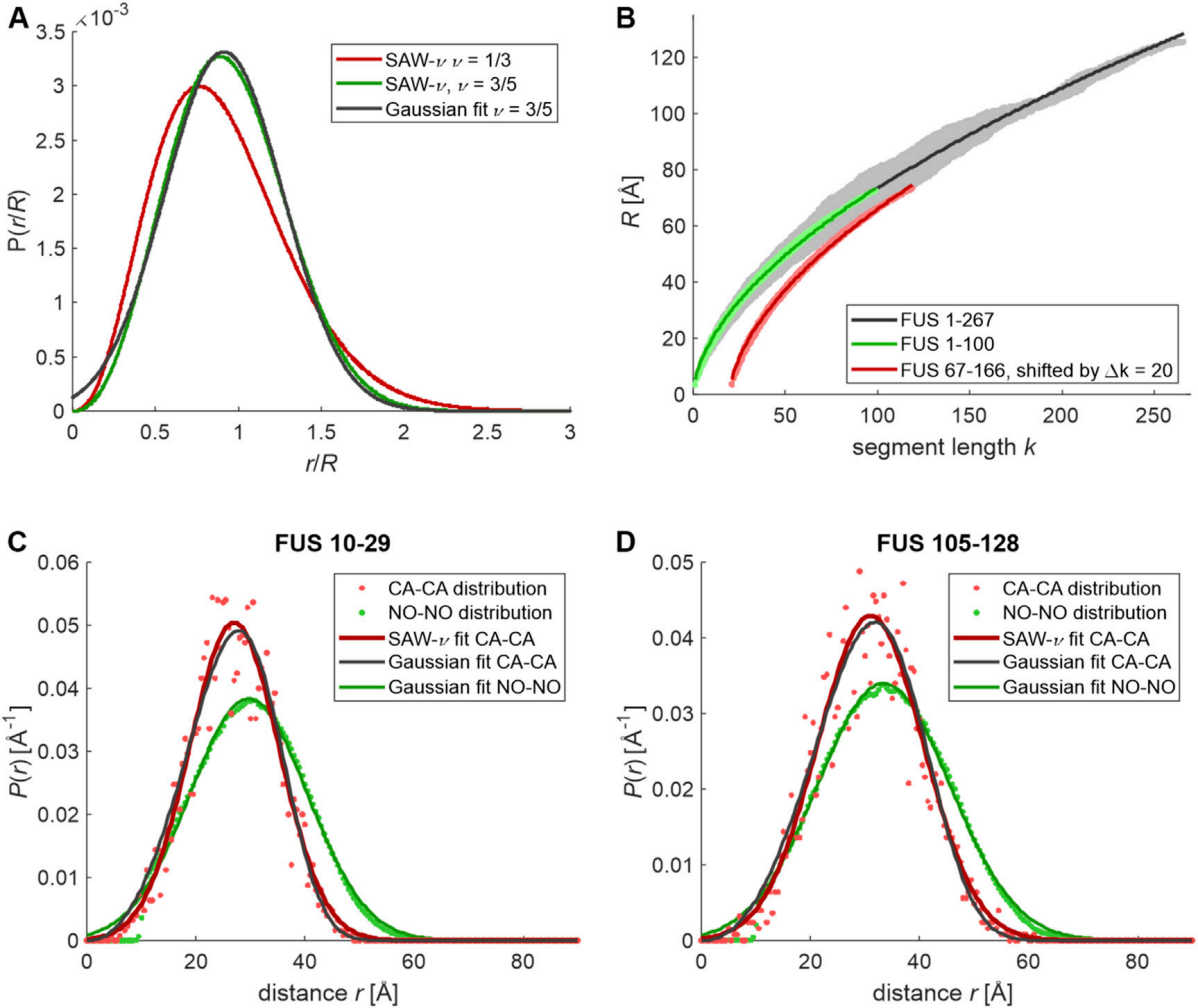
End-to-end and label-to-label distance distributions simulated for the unrestrained NTD of FUS and sections thereof. Residue-specific Ramachandran statistics was assumed as extracted by Hovmoller et al. (2002) from PDB for residues not denoted as helix or strand by DSSP. **(A)** End-to-end distributions predicted by the self-avoid walk model with variable scaling exponent (SAW-ν) by Zheng et al. (2018) for a poor solvent (ν = 1/3, red) and for a good solvent (ν = 3/5, green) and fit of a normal distribution to the good-solvent case (black). **(B)** Segment-wise root mean square CA-CA distances extracted from 90 simulated conformers of FUS 1–267 (gray), and 2,500 conformers of FUS 1–100 (light green) and FUS 66–167 (light red, horizontally shifted by 20 segments for clarity) and fits by a power law *b*·*k* ^ν^ (solid black, green, and red lines, respectively). **(C)** CA-CA distance distribution for residues 10–29 in 5,000 conformers of FUS 1–100 (light red dots) and fits by the SAW-ν model (red line) and a normal distribution (black line) as well as the MTSL-MTSL distance distribution (pale green dots) with fit by a normal distribution (green line). **(D)** CA-CA distance distribution for residues 105–128 in 5,000 conformers of FUS 67–166 (light red dots) and fits by the SAW-ν model (red line) and a normal distribution (black line) as well as MTSL-MTSL distance distribution (pale green dots) with fit by a normal distribution (green line).

In recent work on the NTD of FUS, we found distance distributions between spin labels for the two chain sections 10–29 and 105–128 that were experimentally indistinguishable from a Gaussian distribution, which is symmetric (Emmanouilidis et al., 2021). In particular, we have studied this NTD in a denatured state in the presence of 3 M urea, in a dispersed state at low concentration in the presence of 0.6 M urea, and in a bulk condensed phase that is obtained at 0.6 M urea concentration and higher protein concentration by liquid-liquid phase separation and isolation of the condensed phase by centrifugation. In order to rationalize the finding that distributions are very well approximated in all three cases, we first computed the distributions according to the SAW-ν model for poor- and good-solvent conditions and compared them to a Gaussian distribution (Figure 1A). Whereas in poor-solvent conditions, where the chain maximally compacts (ν=1/3, red line), asymmetry of *P*(*r*) is pronounced, it is only modest in good-solvent conditions (green line). A fit of the good-solvent *P*(*r*) by a Gaussian distribution (black line) shows rather minor deviations that may be hard to resolve by EPR distance distribution measurements. The main deviation at short distances is usually below the lower limit of the distance range of PDS techniques, whereas extension of the SAW-ν distribution to longer distances than in a Gaussian distribution may be hard to separate from intermolecular background.

In order to corroborate this hypothesis, we have generated unrestrained ensembles of the NTD of FUS (residues 1–267) and of two 100-residue sections thereof (1–100 and 67–166) by a Monte-Carlo algorithm that samples from residue-specific Ramachandran distributions of backbone torsion angles for loop regions in proteins (Hovmoller et al., 2002). Otherwise, this algorithm only avoids backbone and sidechain clashes (Jeschke, 2016). The simulation of 100-residue sections allowed to generate much larger raw ensembles than could be obtained within the same computation time for the complete NTD. The ranges were selected such that both studied subsections 10–29 and 105–128 were flanked to both sides with the maximum number of residues possible for a 100-residue construct. We consider the ensuing ensembles as good approximations for the maximum-entropy state of a protein sequence. In order to compare them with the Flory random coil model, which departs from a freely jointed chain model, we analyzed the scaling of the RMS end-to-end distance *R*_*k*_ for all segments with their length *k*, assigning the CA-CA distance between residues *i* and *j* to *k* = *j*-*i* (Jeschke, 2021). For an ensemble of 90 conformers of FUS 1–267 (gray points in Figure 1B), we find a best fit *R*_*k*_ = 5.23·*k* ^0.57^ Å (dark gray line) in rather good agreement with values inferred by FRET and MD simulations for IDPs (Hofmann et al., 2012; Zheng et al., 2018). Variation of the *R*_*k*_ for different pairs (*i*,*j*) at the same *k* appears to be substantial. However, we could trace this back to the low number of conformers used in this analysis. When we repeated the analysis with ensembles of 2,500 conformers each for FUS 1–100 (green) and FUS 67–166 (red), we found a much narrower distribution of the *R*_*k*_ around the best-fit scaling laws 5.25·*k* ^0.57^ Å and 5.17·*k* ^0.58^ Å, respectively. We note, however, that the *R*_*k*_ are not symmetrically distributed around the scaling law at all *k*. The *R*_*k*_ appear to increase somewhat more steeply than the mean scaling law for small *k* and somewhat less steeply for large *k*. This point is extended below.

We have then inquired whether the distributions of CA-CA distances in the unrestrained ensembles (red dots in Figures 1C,D) conform to the SAW-ν distribution (red lines). We find *R* = 27.7 Å and ν = 0.78 with a RMS deviation (RMSD) of 0.163 for section 10–29 in FUS 1–100 and *R* = 31.9 Å, ν = 0.77 with RMSD 0.171 for section 105–128 in FUS 67–166. The scaling exponents ν appear to be too large, as they should not exceed 0.6 for a random coil in a good solvent. Moreover, Gaussian fits (black lines) of the same distributions result in similar RMSD. We find a mean value ⟨*r*⟩ = 27.0 Å and standard deviation σ_*r*_ = 8.2 Å with RMSD 0.155 for section 10–29 in FUS 1–100 and ⟨*r*⟩ = 31.0 Å and standard deviation σ_*r*_ = 9.7 Å with RMSD 0.170 for section 10–29 in FUS 105–128.

Measurement of the distribution between spin labels further complicates detection of the asymmetry, as the label-to-label distribution is a convolution of the CA-CA distribution with a contribution from the sidechain rotamer distribution of the label. In order to assess this effect, we have simulated the label-to-label distribution by a rotamer library approach (Polyhach et al., 2011) in MMM (Jeschke, 2018). Since on the order of 100 × 100 rotamers at the two sites are populated for each backbone conformer, the resulting distributions (green points in Figures 1C,D) are much smoother. They are fitted quite well by Gaussian distributions (dark green lines) with ⟨*r*_label_⟩ = 29.7 Å and standard deviation σ_*r*,label_ = 10.8 Å for section 10–29 in FUS 1–100 and ⟨*r*_label_⟩ = 33.4 Å and standard deviation σ_*r*,label_ = 12.1 Å for section 105–128 in FUS 67–166.

**FIGURE 2:**
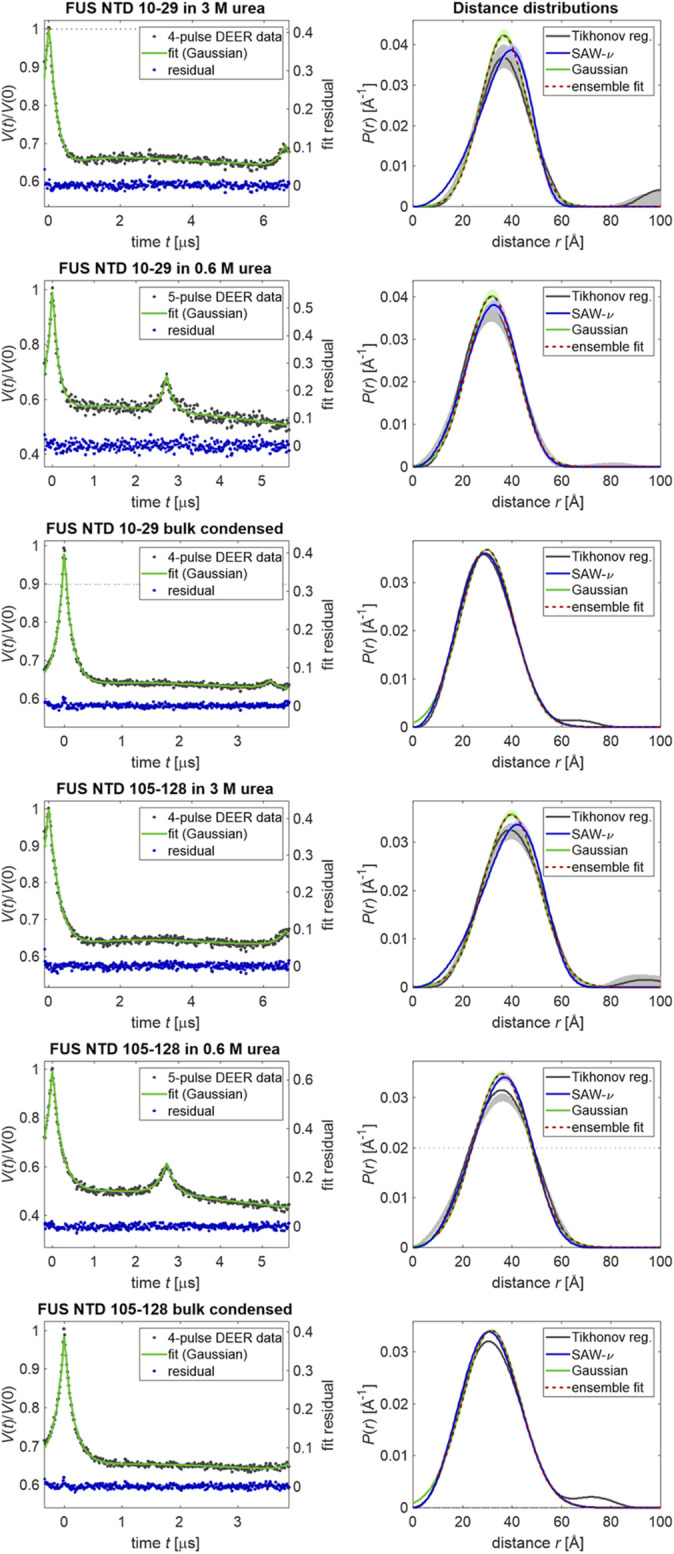
Distance distribution measurements of sections delimited by spin labeling sites (10–29 or 105–128) measured with FUS NTD 1–267. The left column shows primary data (black dots), their DeerLab fit by a single Gaussian distribution (green, left ordinate) and the fit residual (blue dots, right ordinate). Signals from minor pathways at the end of 4-pulse DEER data or at the middle of 5-pulse DEER data were included in the kernel (Fabregas Ibáñez et al., 2020). The right column shows distance distributions obtained by Tikhonov regularization with choice of the regularization parameter by the Bayesian information criterion or residual method (black with gray uncertainty band), by single-Gaussian fitting (green with pale green uncertainty bands) and ensemble fits of the Gaussian distribution (red) from raw ensembles of 2,500 conformers each.

Figure 2, with original data taken from (Emmanouilidis et al., 2021), demonstrates that the quality of Gaussian distribution fits to the primary DEER data is high for FUS 1–267 denatured in 3 M urea, fully dispersed at 5 μM protein concentration in 0.6 M urea, and in bulk condensed phase after LLPS. In the left column, primary data that were only phase and zero-time corrected (black dots) are superimposed by fits in DeerLab (Fabregas Ibáñez et al., 2020) in which a single Gaussian distance distribution and an exponential background function are simultaneously fitted (green line, left ordinate). The exponentially decaying background accounts for interaction with spin labels in other FUS NTD molecules that are assumed to be homogeneously distributed in three-dimensional space. The residuals are shown as blue dots (right ordinate). The only apparent deviations of the residual from the expected white noise occur near zero time in the two bulk condensed phase measurements and correspond to an underestimate of the short distance contribution by the fit. Note that an overestimate would be expected if the data were better represented by an SAW-ν distribution. The likely cause of the underestimate is neglect of the contribution at very short distances in the DeerLab simulations, as it is expected to be suppressed by insufficient excitation bandwidth. This suppression appears to be weaker than assumed.

In the right column of Figure 2, distance distributions obtained by Tikhonov regularization (black lines with gray uncertainty bands, 95% confidence interval) are compared with the fitted Gaussian distributions (dark green lines with pale green uncertainty bands). Tikhonov regularization does not make an assumption about the shape of the distribution, except for a certain degree of smoothness. Although it can be argued that the Tikhonov distributions deviate from the Gaussian distributions toward the expected more asymmetric shape with lower contributions at short distances and higher contributions at the longest distances, these deviations are minor and are clearly seen only in the bulk condensed phase data. Nevertheless, we have attempted fits of the DEER data by the SAW-ν model. The fitted distributions are shown as blue lines with pale blue uncertainty bands in Figure 2, the fit parameters are reported in Table 1 alongside the parameters of the Gaussian fits, and the fits of the primary data and fit residuals are shown in Supplementary Figure S1. Only for FUS 1–267 in 3 M urea do the SAW-ν fits differ significantly from the distributions obtained by Tikhonov regularization or Gaussian fits. However, in these cases the asymmetry is opposite to the expected one, with more gradual increase of *P*(*r*) at short distances and steeper decrease at longer distances. This can be traced back to the unphysical scaling exponents of ν = 0.85 for segment 10–29 and 0.82 for segment 105–128 that we find by these fits. These findings reinforce our conclusion that parameters of the SAW-ν model cannot reliably be extracted by fitting DEER distance distributions for chain segments containing about 20 residues. The maximum-entropy reference state is well approximated by Gaussian label-to-label distance distributions. On the one hand, this finding justifies the use of Gaussian restraints in generating raw ensembles, as introduced in (Jeschke, 2016). On the other hand, it suggests that longer segments would need to be studied in order to extract parameters of the SAW-ν model. Such an approach would, however, be limited by uncertainty of the shape of DEER distance distributions at distances longer than 50–80 Å.

**TABLE 1:** Fit parameters of experimental distance distributions for MTS labels attached at the ends of segments 10–29 and 105–128 in FUS NTD 1–267. The mean value ⟨*r*_DEER_⟩ and standard deviation σ_*r*,DEER_ are specified for Gaussian fits, while the RMS end-to-end distance *R*_DEER_ and scaling exponent ν_DEER_ are specified for the SAW-ν distribution.

Site1	Site 2	Conditions	⟨r_DEER_⟩ [Å]	σ_*r*,DEER_ [Å]	*R*_DEER_ [Å]	ν_DEER_
10	29	3 M urea	36.7	9.4	37.4	0.85
10	29	0.6 M urea	31.8	9.9	33.2	0.75
10	29	Bulk condensed	29.7	10.9	31.8	0.64
105	128	3 M urea	39.7	11.2	40.9	0.82
105	128	0.6 M urea	35.9	11.5	37.3	0.76
105	128	Bulk condensed	31.6	11.7	33.7	0.64

We also note that measurements for the characterization of LLPS are more sensible in the biphasic system containing both the dispersed and condensed phase under otherwise identical conditions, as such samples match the surface-to-volume ratio of biological membraneless organelles and such measurements require much less protein (Emmanouilidis et al., 2021). In such systems, however, the separation of the distance distributions corresponding to the dispersed and condensed phase introduces further uncertainty. We have therefore limited our analysis in the present study to the distance distributions of monophasic protein samples (either in solution or a large phase-separated compartment).

#### A Hybrid Experimental and Computational Approach

We have then turned to the question whether a hybrid experimental/computational approach can shed more light on the reference state. To that end we have generated six raw ensembles of 2,500 conformers each of FUS 1–100 and 67–166 by the Monte Carlo approach introduced in (Jeschke, 2016) by imposing the Gaussian restraints for sections 10–29 and 105–128, respectively, in the denatured, dispersed, and bulk condensed states. We have then refined these ensembles by fitting populations of the conformers and discarding all conformers with less than 1% of the population of the most populated conformer, as described in (Jeschke, 2021). These computations have been performed with an implementation of the EnsembleFit module of MMM into the successor program MMMx^2^. With this size of the conformer basis set, we find that the Gaussian distance distributions can be fitted virtually perfectly for both sections in all conditions tested (red dashed lines in the right column of Figure 2). The overlap of the normalized experimental and ensemble-simulated distance distributions varies between 98.2% (105–128, 0.6 M urea, dispersed) and 99.1% (10–29 0.6M urea, dispersed) with sizes of the refined ensemble between 47 (10–29, 3 M urea, denatured) and 145 (10–29, 0.6 M urea, dispersed) conformers. We found that fit quality was converged with respect to adding further conformers from the raw ensemble after evaluating about 1,000 conformers. We conclude that the methodology of generating raw ensembles of conformers by a Monte Carlo approach and refining and reducing these ensembles by population fitting can provide relatively small ensembles that fully represent the information contained in EPR-measured label-to-label distance distributions.

**FIGURE 3:**
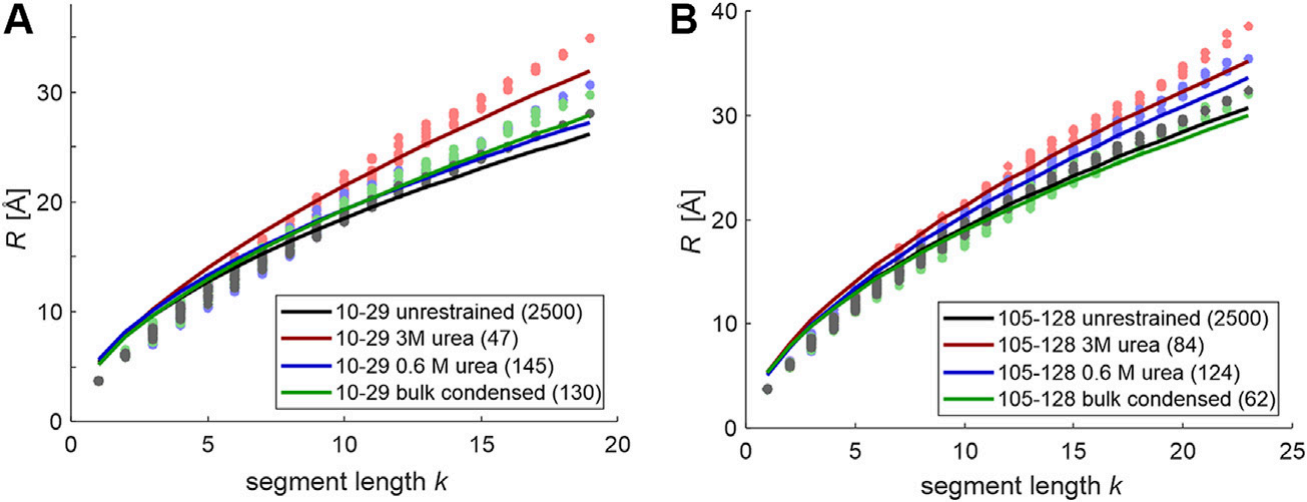
Segment-wise root mean square CA-CA distances (dots) for chain sections 10–29 in FUS 1–100 **(A)** and 105–128 in FUS 67–166 **(B)** and attempted fits by a scaling law *b*·*k* ^ν^. For unrestrained ensembles, the average is over 2,500 conformers with uniform population. For restrained ensembles, the average population-weighted and the number of conformers in the fitted ensembles is given in the legend in parentheses.

We have then tested whether these ensembles allow for inference on the scaling exponent ν of random-coil models. To that end, we have performed the analysis of the scaling of *R*_*k*_ for all segments of length *k* for FUS sections 10–29 and 105–128 for all conditions tested. The data is displayed in Figure 3 together with data from the unrestrained ensembles (gray). In all cases we find that fits by a scaling law *b*·*k* ^ν^ are mediocre. Fitted scaling exponents ν are in a reasonable range between 0.538 (FUS 10–29 unrestrained) and 0.619 (FUS 10–29, 3M urea), as are the Kuhn lengths *b* between 5.14 Å (FUS 105–128, 0.6 M urea) and 5.58 Å (FUS 10–29, 0.6 M urea). However, deviations from the fits are systematic, with longer RMS end-to-end distances for the longest segments than predicted. This corresponds to chains that are stiffer than predicted. Comparison of the unrestrained case with Figure 1B reveals that such deviations are much smaller for longer chains than for the short sections considered here. Such behavior of shorter chain sections being stiffer than longer ones we have encountered before in the context of semi-rigid organic polymers (Godt et al., 2006; Jeschke et al., 2010). We note that the sections studied here are oligomers in the sense of the Flory random-coil model, as their length is less than ten times the Kuhn length *b*. As a consequence, we caution against assuming random-coil behavior for the fully disordered reference state. Instead, we advocate unrestrained Monte-Carlo simulations of large ensembles of conformers based on Ramachandran statistics for loop regions. These raw ensembles can then be fitted to experimental distance distributions and the resulting refined ensembles can be analyzed in terms of the distributions of *R*_*k*_ for segment lengths *k*. If these distributions are narrow and scaling of the mean values is monotonous and smooth, we can assume the chain to be in a fully disordered state. This need not be the case in general, as we demonstrate in the following on the example of the glycine-rich domain of hnRNP A1. This domain has high composition similarity to the FUS NTD, but was here studied in the context of the full length protein including the folded RNA-recognition motifs.

### Computation of Distance Distributions for Ensemble Fitting

#### Choice of DEER Data Processing Approach

Accuracy of DEER distance distributions is limited by experimental and computational aspects (Jeschke, 2012). From an experimental point of view, excitation bandwidth limitations cause partial suppression of contributions below about 18 Å. Since the assumptions of weak coupling of the two spins and of negligible exchange coupling break down below about 15 Å, few efforts have been made to overcome this excitation bandwidth limitation. At long distances, the shape or even width of the distribution become uncertain, as this information is encoded in the decay of the dipolar oscillations and can thus be recovered only up to distances where several dipolar oscillation periods are still within the maximum observation time *t*_max_. The accessible *t*_max_ depends on electron spin decoherence time and can vary strongly between proteins. For soluble proteins measured with high-power Q-band DEER, shape information is typically reliable up to 50 to 80 Å, depending on whether the solvent and protein can be deuterated. The reliable distance range for a given sample also depends on the signal-to-noize ratio and on the presence of instrumental or experimental artifacts in the data, as conversion of the primary data to a distance distribution is an ill-posed problem where a least-squares solution is strongly affected by slight deviations of the data from the forward model (Jeschke et al., 2002; Chiang et al., 2005). During the past two decades, several approaches were suggested for tackling this problem, such as Tikhonov regularization (Bowman et al., 2004; Jeschke et al., 2004; Chiang et al., 2005), fitting by multiple Gaussians, whose number is determined by a statistical criterion (Stein et al., 2015), and training an ensemble of neural networks with large sets of forward-modelled data that cover the expected men values, widths, and shapes of DEER signals (Worswick et al., 2018). All recent implementations provide, both, the most probable distance distribution according to the computational approach and confidence intervals. We note, however, that estimates of confidence intervals for ill-posed problems may not cover model bias, which for regularization approaches includes bias arising from the choice of the regularization parameter. The latter aspect has been studied recently (Edwards and Stoll, 2018) and different criteria have been proposed for the choice of the regularization parameter. With such a large variety of approaches existing, it is of interest whether ensemble modeling is robust with respect to the choice of the data processing approach. The problem is further complicated by the necessity to separate the dipolar evolution function from intermolecular background (Jeschke et al., 2006). Analysis of the DEER data for FUS NTD sections above has revealed that different fit approaches may lead to different distance distributions even for data sets of rather high quality.

In our approach, we first generate a raw ensemble of conformers with restraints that specify only the mean and standard deviation of a single Gaussian per site pair (Jeschke, 2016). These parameters are more robust than the shape of the distribution (Jeschke et al., 2004; Jeschke, 2012). In a second step, we reweight and contract the raw ensemble by varying conformer populations and discarding conformers with less than 1% of the population of the most populated conformer (Jeschke, 2021). In this ensemble fitting step, we use full distance distributions rather than only mean distance and width. However, even if the distribution used in the second step is the same Gaussian used in the first step, ensemble reweighting can improve the fit quality with the same or with an even smaller number of conformers. Thus, the question arises which approach should be used for generating distance distributions for the use as restraints in ensemble reweighting, which depends on the selected fit criterion. Here, we consider maximization of overlap of experimental and predicted distance distributions (Jeschke, 2021), defined by

$$o_{d}=\sum \min\left\{P_{pred},P_{DDR}\right\},$$ (1)

where ***P***_pred_ is the forward-modelled distance distribution for the reweighted ensemble and ***P***_DDR_ is the distance distribution obtained from experimental data, both given as vectors with non-negative elements whose sum is unity.

In particular, we maximize the geometric mean $\bar{o}={\left(\overset{M}{\underset{m=1}{\prod }}o_{m}\right)}^{1/M}$ of the overlaps *o*_*m*_ for all *M* restraints, which strongly penalizes large overlap deficiency 1- *o*_*m*_ of individual restraints. Even if the ensemble of conformers is consistent with the primary time-domain data, large overlap deficiency can arise if conversion to the distance distribution uses a poorly suited approach. In principle, the problem could be solved by directly fitting to the primary data (Hilger et al., 2007). However, this makes fitting computationally expensive and would preclude global optimization of populations (Jeschke, 2021) for large sets of conformers. An alternative ensemble reweighting approach based on the maximum entropy principle (Köfinger et al., 2019) could be used as well in this second step.

In order to assess current approaches for conversion of dipolar signals to distance distributions in this context, we have analyzed 19 DEER data sets obtained on dispersed hnRNP A1. Given that there is no prior structural information available for a priori screening of suitable labeling in the LCD, we primarily mutated available Ser residues with approximately uniform sequence separations to Cys. The labeling sites in the folded domains of hnRNP A1 were selected to be spatially well separated and solvent accessible. Note that label site selection in folded domains typically requires case-by-case considerations with general guidance provided by rotamer modeling (Polyhach and Jeschke, 2010). Specifically, for the generation of distribution restraints labeling sites in folded domains with low local backbone flexibility can provide more precise distance information (Jeschke, 2016), but may be less tolerant toward site-directed mutagenesis and successive spin labeling. The selected sites 52 and 144 are located in partially flexible loops of the RRMs of hnRNP A1, which provides a good trade-off. The dataset consists of 16 restraints between RRM-LCD sites, and three between LCD-LCD sites.

**FIGURE 4:**
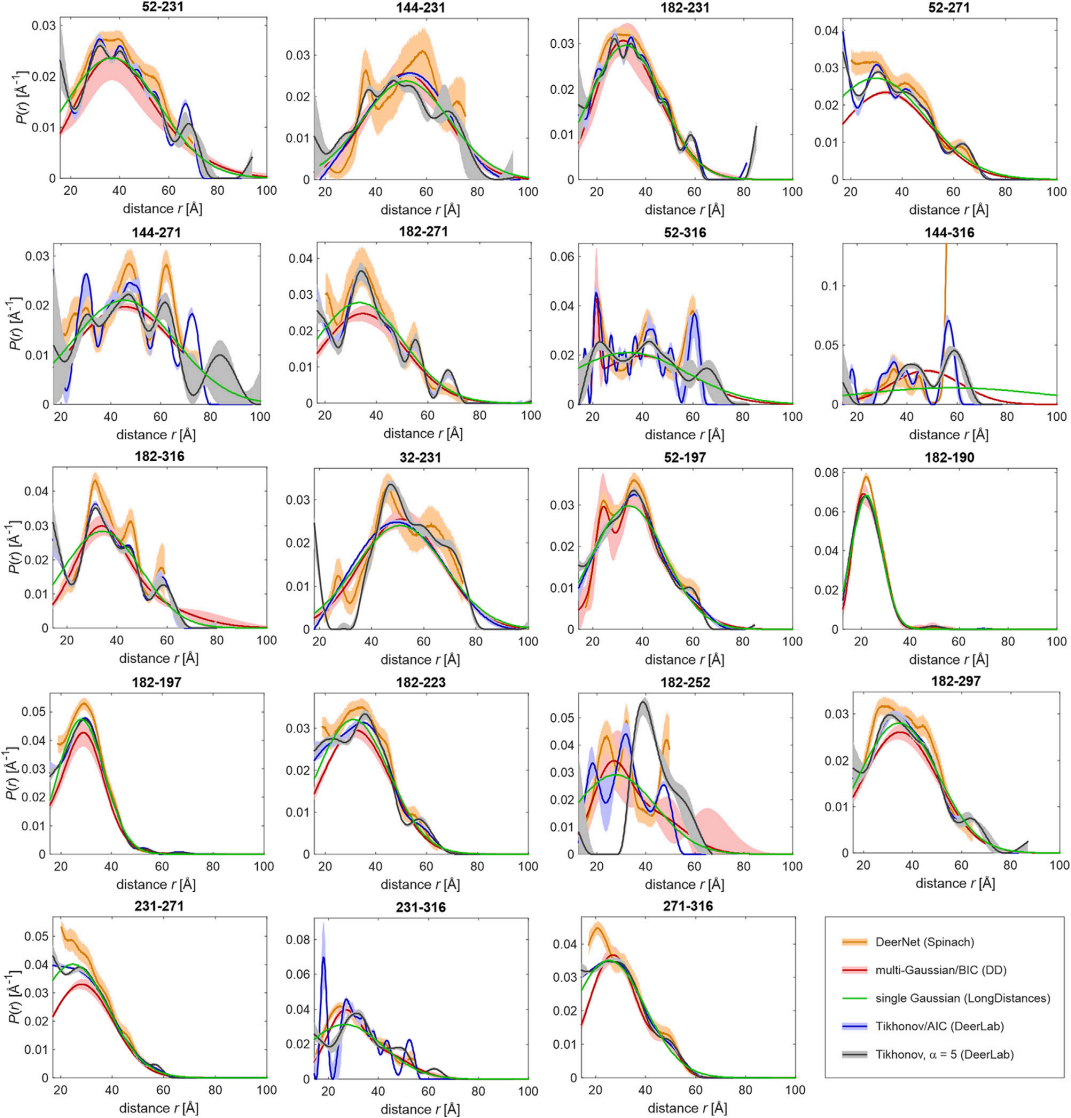
Distance distributions computed by different approaches from primary DEER data for 19 pairs of MTS-labelled sites. The approaches are DeerNet in Spinach 2.5.5446 (orange), multi-Gaussian fit with the number of Gaussians determined by the Bayesian information criterion (BIC) in DD (red), single Gaussian fit including background in LongDistances (green), Tikhonov regularization with regularization parameter α determined by the Akaike information criterion (AIC) in DeerLab 0.8b (blue), and Tikhonov regularization with fixed α = 5 in DeerLab 0.8b (gray/black). Pale areas denote uncertainty bands (50% confidence interval for DeerLab, 95% confidence interval for DeerNet and DD).

The data were analyzed by fitting with a single Gaussian distribution, multiple Gaussian distributions, whose number is determined by a statistical criterion (Stein et al., 2015), the neural network approach DeerNet (Worswick et al., 2018), and Tikhonov regularization (Bowman et al., 2004; Jeschke et al., 2004; Chiang et al., 2005) with the regularization parameter either determined by a statistical criterion (Edwards and Stoll, 2018) or fixed at a value that is judged from appearance of the distributions (Figure 4). Multi-Gaussian fitting was performed in DD (Stein et al., 2015) using either the Akaike or Bayesian information criterion for determining the optimal number of Gaussian components. Analysis in terms of a single Gaussian distribution was performed with three different software packages, DeerAnalysis (Jeschke et al., 2006) version 2019, DeerLab (Fabregas Ibáñez et al., 2020) version 0.8b, and LongDistances^3^. The latter two programs simultaneously fit the distance distribution and background, whereas in DeerAnalysis we first performed background separation and then fitted the dipolar evolution function. Figure 4 shows the results obtained with LongDistances. The mean distances and standard deviations obtained with all three approaches are listed in Table 2. For most data sets, agreement between the results of all three approaches is rather good, with the exceptions of site pairs 144–316 and 182–252. In these two cases, the single-Gaussian model is too simplistic for separating the pair contribution from background with sufficient certainty. As an example for fit quality of the primary data, the results obtained with Tikhonov AIC fitting are shown in Supplementary Figure S2.

**TABLE 2:** Single-Gaussian restraint sets (mean distance ⟨*r*⟩ and standard deviation σ_*r*_) for hnRNP A1 obtained with three different program packages (DeerAnalysis, DA; DeerLab, DL; LongDistances, LD).

Site1	Site2	⟨*r*⟩_DA_ [Å]	*σ*_DA_ [Å]	⟨*r*⟩_DL_ [Å]	*σ*_DL_ [Å]	⟨*r*⟩_LD_ [Å]	*σ*_LD_ [Å]	⟨*r*⟩_DL_/⟨*r*⟩_DA_	⟨*r*⟩_LD_/⟨*r*⟩_DA_
52	231	37.5	18.1	36.7	19.5	36.9	19.4	0.979	0.984
144	231	49.7	15.4	52.7	14.9	51.8	17.2	1.060	1.042
182	231	33.3	14.8	31.7	14.8	31.7	14.8	0.952	0.952
52	271	28.1	19.7	29.4	19.9	29.5	19.9	1.046	1.050
144	271	45.7	18.6	44.7	21.1	45.2	20.8	0.978	0.989
182	271	30.5	18.8	33.2	17.1	33.4	17.2	1.088	1.095
52	316	37.5	18.2	36.3	21.2	34.2	23.6	0.968	0.912
144	316	44.1	11.7	45.6	16.9	57.3	38.5	1.034	0.873
182	316	34.8	15.7	33.8	15.6	34.0	15.6	0.971	0.977
32	231	51.2	15.5	50.7	15.8	50.9	17.1	0.990	0.994
52	197	35.6	13.8	34.9	14.2	34.4	14.5	0.980	0.966
182	190	22.5	6.2	22.0	6.3	22.2	6.1	0.978	0.987
182	197	27.4	9.5	27.9	9.2	28.1	9.1	1.018	1.026
182	223	31.4	14.2	31.0	14.5	31.1	14.2	0.987	0.990
182	252	36.6	12.7	29.9	15.1	27.6	16.5	0.817	0.754
182	297	34.1	15.8	34.2	16.1	34.5	15.9	1.003	1.012
231	271	20.1	15.3	24.5	14.1	24.7	13.9	1.219	1.229
231	316	27.1	16.5	28.5	14.1	26.5	15.4	1.052	0.978
271	316	22.6	15.8	24.9	14.6	25.3	14.5	1.102	1.120

Turning to different distribution models, for site pair 144–316 with a very broad distance distribution extending to rather long distances, all approaches run into difficulties, although agreement between Tikhonov regularization, DeerNet and multi-Gaussian fitting is reasonable. Splitting of a broad distribution into many moderately broadened peaks, such as encountered for site pair 52–316 when using the Akaike information criterion for determining the regularization parameter for Tikhonov regularization, may be detrimental to fitting with the overlap criterion as it substantially reduces overlap (Supplementary Figure S3 left). For this reason, we have resorted to Tikhonov regularization with a fixed regularization parameter α = 5 (Supplementary Figure S3, middle), which is not expected to oversmooth distributions for the case at hand. In contrast, a single narrow Gaussian component, as it is sometimes found with the multi-Gaussian parametrized model, is less detrimental, since it contributes only a small fraction of the distribution (Supplementary Figure S3, right).

For most of the site pairs, agreement of the distance distributions is good between all approaches. This applies, in particular, to comparatively narrow distributions, which do not extend beyond 60 Å. For data sets where the approaches disagree more strongly, we also find broader uncertainty bands with the individual approaches. Note, however, that not all distributions agree within their specified uncertainties. Differences between the distance distributions obtained with different approaches indicate limited quality of the data sets, mainly because of mediocre signal-to-noize ratio or a maximum observation time that is insufficient for high confidence in background separation. We note that these features are not a sign of poor experimentation. Depending on width of the distribution and maximum distance, obtaining higher-quality data may be unrealistic.

#### Ensemble Fitting to Distance Distributions With Moderate Shape Uncertainty

The question then arises, whether distance distributions of such site pairs can still be used in ensemble fitting. The answer is not necessarily negative, since the overlap metric is less affected by the differences than the appearance of the distributions. Therefore, we have studied this question in detail. To that end, we have performed ensemble fitting from the same raw ensemble for all individual sets of distance distributions displayed in Figure 4, as well as for the Gaussian distribution obtained by DeerLab (parameters listed in Table 2). As a result, we have obtained refined ensembles with a reduced number of conformers and with populations assigned to these conformers.

**FIGURE 5:**
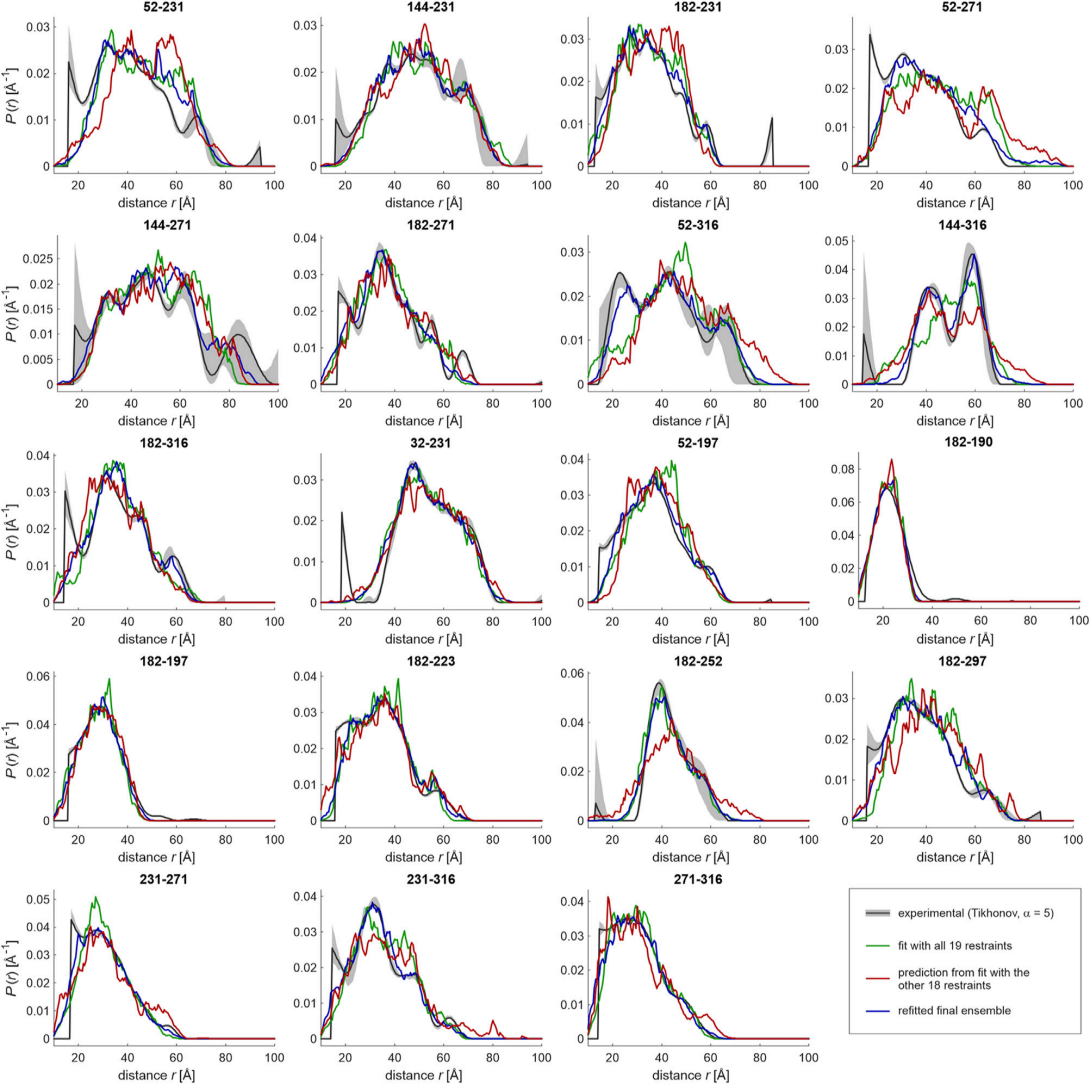
Ensemble fitting of the LCD (residues 188–320) of hnRNP A1 with jack-knife resampling. A raw ensemble of 331 conformers was generated based on Gaussian restraints. Conformer populations were fitted by maximizing geometric mean overlap between simulated (green) and experimental (black) distance distributions. Conformers with less than 1% of the population of the most populated conformer were discarded, resulting in a refined ensemble with 60 conformers. The procedure was repeated with all possible sets of 18 out of the 19 restraints, giving slightly different sizes of the raw and refined ensembles. The unused restraint was predicted from the “leave-one-out” ensemble (red). All conformers found in individual ensembles during jack-knifing were combined with the 60 conformers in the initial ensemble and used as the basis set for the final ensemble fit (blue lines).

The overview of results in Table 3 demonstrates that fit quality and size of the population-fitted ensembles vary only moderately between the different restraint sets, despite the apparent differences in the fitted distance distributions (i.e., the resolution of spikes and peaks) obtained with the different approaches (see Figure 4). As an example for fit quality, results are shown in Figure 5 for the case of Tikhonov regularization with fixed regularization parameter α = 5, with the experimental distributions as black lines with gray uncertainty bands and the prediction from the fitted ensemble as green lines. We note that in some cases, features of the distribution shape are fitted that cannot be reproduced by a Gaussian, most notably for site pairs 32–231, 182–223, and 182–252. Thus, reweighting of conformers after generation of the raw ensemble can partially recover underfitting of structural information in the input single Gaussian model. Still, geometric mean overlap $\bar{o}$ is maximum for the ensemble fit to the single Gaussian distributions obtained in DeerLab.

**TABLE 3:** Geometric mean overlap $\bar{o}$, number of conformers, radius of gyration *R*_g_, and ensemble width Γ in ensembles of hnRNP A1 LCD (188–320) by using distance distributions computed with different approaches (see also Figure 4) in ensemble fitting from the same raw ensemble.

Analysis approach	Mean overlap $\bar{o}$	Number of conformers	*R*_g_ [Å]	Γ [Å]
DeerNet	0.836	57	24.0	46.6
Multi-Gaussian (AIC)	0.880	52	23.3	47.6
Multi-Gaussian (BIC)	0.882	56	23.4	47.6
Single Gaussian (LongDistances)	0.863	49	23.7	48.2
Single Gaussian (DeerLab)	0.883	52	23.7	47.4
Tikhonov (α by AIC criterion)	0.846	50	23.8	48.4
Tikhonov (α = 5)	0.876	50	23.7	48.2

**FIGURE 6:**
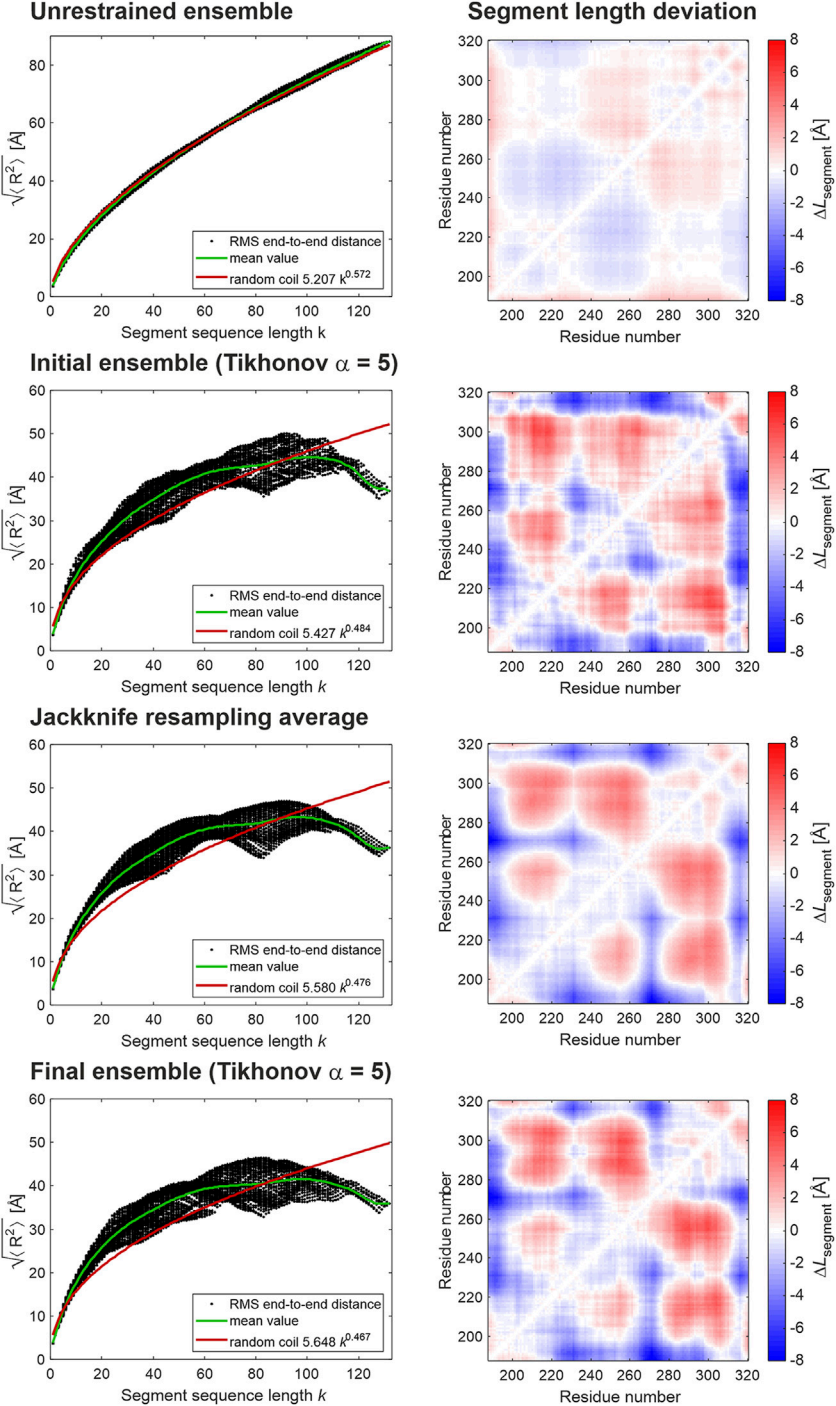
Ensemble analysis for the LCD of hnRNP A1. The left column shows root mean square CA-CA distances for all possible segments of the LCD (residues 188–320) as a function of segment sequence length (black dots), their mean values per segment sequence length (green line), and a random coil fit (red line). The right column shows the deviation of the RMS CA-CA distances from the mean value for this segment sequence length (green line in the left column). Red hues correspond to segments more extended than the average and blue hues to segments more compact than the average.

We have also compared the ensembles obtained with restraints sets from the six data analysis approaches. Such comparison is complicated by the fact that the sets of selected conformers are distinct and the domain is only moderately structured. Therefore, we have opted for ensemble analysis approaches that can reveal weak structure. First, we considered segment-wise RMS end-to-end distances (Jeschke, 2020) as displayed for FUS in Figures 1B, 3. The corresponding plots for hnRNP A1 LCD are shown in the left column of Figure 6. Whereas the unrestrained ensemble (top) exhibits similar behavior as the FUS NTD ensembles, for the restrained ensemble model of hnRNP A1 (second from top), scaling of segment end-to-end distances is less regular and the distributions are broader. While the patterns obtained with different data analysis approaches (Supplementary Figures S4, S5, left column) are not identical, they do exhibit very similar features. In particular, with all approaches the LCD of hnRNP A1 exhibits scaling behavior that decidedly differs from the one of the unrestrained ensemble. In the restrained ensembles, segment-wise end-to-end distances do not increase monotonously, but rather decline at segment sequence lengths *k* > 100. Furthermore, segment compactness varies considerably at the same sequence length in the range 40 < *k* < 100. In order to analyze this variation in more detail, we use a measure related to the proximity matrix introduced in (Jeschke, 2021). The proximity matrix is related to, but distinct from, the contact matrix or map that can be obtained by NMR techniques, such as paramagnetic relaxation enhancement (Salmon et al., 2010; Clore, 2013). Unlike the contact map, which reveals close approach of residues with large sequence distance, the proximity matrix quantifies the deviation of the RMS CA-CA distance of residue pairs from the one predicted for that segment length *k* from the random-coil scaling law *b*·*k* ^ν^, where parameters *b* and ν are fitted to all segments of the chain. This deviation is normalized to the predicted RMS CA-CA distance. Thus, the proximity matrix also reveals compaction of chain segments that does not lead to contact. For the case at hand, we consider a more intuitively interpretable matrix **Δ*L***, whose elements are the absolute deviation of the RMS CA-CA distance of residue pairs (*i*,*j*) from the mean RMS CA-CA distance for all segments of the same length *k* (green line in the right column in Figure 6). The matrix elements Δ*L*_*ij*_ for residue pairs (*i*,*j*) are then defined as

$$\text{Δ}L_{ij}=\sqrt{\langle R_{ij}^{2}\rangle }-\frac{1}{n_{k}}\sum_{m-l=k}\sqrt{\langle R_{lm}^{2}\rangle }$$ (2)

where *n*_*k*_ is the number of residue pairs (*l*,*m*) with *k* = *j*–*i* = *m*–*l* and 〈⋯〉 denotes the ensemble average. This matrix is visualized in Figure 6 (right column) with blue color encoding segments shorter than average and red color encoding those longer than average. The color scale is the same for all ensembles.

In the restrained ensembles (second row from top), we find a pattern of locally shortened and lengthened segments that is broadly replicated with all distance analysis approaches (right column in Supplementary Figures S4, S5), although details differ. This pattern strongly differs from the unrestrained reference state (top row), which exhibits behavior that is very similar to the one of the reference state for FUS NTD.

Likewise, radii of gyration of the LCD are very similar (minimum 23.3 Å for multi-Gaussian distributions, maximum 24.0 Å for DeerNet) as are ensemble widths

$$\text{Γ}=\sqrt{\frac{\sum_{i=1}^{N-1}\sum_{j=i+1}^{N}p_{i}p_{j}D_{ij}^{2}}{\sum_{i=1}^{N-1}\sum_{j=i+1}^{N}p_{i}p_{j}}}$$ (3)

where indices *i* and *j* run over conformers in the ensemble, the *p*_*i*_ and *p*_*j*_ are conformer populations, and the *D*_*ij*_ are RMS coordinate deviations between conformers upon optimal superposition. We find that Γ varies between 46.6 Å and 48.4 Å (Table 3). In conclusion, ensemble fitting to distance distributions by maximization of overlap is rather robust with respect to moderate variation in distribution shape.

### Validation of the Restraint Set by Jack-Knife Resampling

For atomic-resolution structures, it is relatively well understood what constitutes a good or at least a sufficient restraint set. The same cannot be said for ensemble modeling of weakly structured proteins. On the one hand, the problem appears hopelessly underdetermined. Backbone conformation of each individual conformer with *n*_res_ residues is determined by 2(*n*_res_-1) torsion angles, which would suggest 262 free parameters per conformer for the glycine-rich domain of hnRNP A1 (188–320), whereas we have obtained only 19 distance distribution restraints. On the other hand, the unstructured reference state is characterized by only one (radius of gyration *R*_g_ or RMS end-to-end distance *R*) or at most two (*b*, ν) parameters. As we do not know beforehand, how strongly a particular protein or domain is structured, we need to estimate the number of required restraints during modeling. Moreover, the restraint set might not be internally consistent. With labeling approaches, this may happen if a label biases conformation. We have encountered such a case for the FnIII-3,4 domains of integrin α6β4, were some spin-labelled mutants had to be discarded, as they caused strong changes in relative domain orientations as seen by changes in small-angle scattering (SAXS) curves (Alonso-García et al., 2015). In a computational study on amyloid-beta, it was found that attachment of a spin label biased the conformer distribution of this intrinsically disordered peptide to some extent (Sasmal et al., 2017). It is not always possible to safely exclude such bias by additional experiments. Hence, size and internal consistency of a set of distance distribution restraints need to be validated alongside modeling.

Robustness of an ensemble can be estimated by resampling approaches, such as bootstrapping or jack-knifing (Berman et al., 2019). In jack-knifing, as many additional modeling runs are performed as there are restraints. In each run, one of the restraints is left out without replacement. It is tested, how strongly the ensemble changes and how well the left out restraint is predicted. Jack-knife resampling has been used before in the context of distance distribution restraints for estimating uncertainty of a high-resolution model of the dimer of Na^+^/H^+^ antiporter NhaA (Hilger et al., 2007). Bootstrapping approaches often remove more than one restraint and they replace left-out restraints by some remaining restraints, effectively increasing the weight of these doubly selected restraints.

Here, we implement jack-knife resampling in our ensemble modeling pipeline. To that end, we have generated raw ensembles of about 400 conformers each for all 19 restraint sets where one of the restraints is left out. We have then performed ensemble fitting with the same restraint left out and predicted the distance distribution for this unused restraint. These 19 predictions from 19 ensemble fitting runs are displayed as red lines in Figure 5. In general, they agree quite well with the experimental distributions, indicating that the set is, both, internally consistent and sufficiently large. Interestingly, while several restraints were measured as permutations of label combinations of major reporter sites, a few sites appeared only in a single restraint (folded: 32; LCD: 190, 197, 223, 252, and 297), and might thus be more critical for overall ensemble convergence. Indeed, the case of 182–252 is such a case of an ‘isolated’ restraint for which the deletion appears to lead to a slightly broader ensemble compared to the fit with all restraints. However, the predicted restraint fulfilment upon deletion of a restraint was not in general worse for isolated restraints than for restraints involving multiply restrained sites. Therefore, at least in this particular case of hnRNP A1 it appears that the coverage of labeling sites in the LCD sequence was sufficient to avoid modeling bias. However, it is clear that such consideration must be made case-by-case, since the problem is strongly dependent on geometry and extent of ordering of domains in a given protein (Jeschke, 2016).

Returning to hnRNP A1, in a few cases, notably for site pairs 52–271, 52–316, 144–316, and 182–252, background separation may have removed genuine contributions at long distances. Except for pair 52–271, these distributions were flagged as being less reliable by disagreement between results from different data analyses approaches. In a few other cases, such as 182–271 and 32–231, the leave-one-out predictions appear to confirm distance distribution shapes that deviate from a single Gaussian.

We have combined all 19 leave-one-restraint-out ensembles and have renormalized populations to unity sum. From this “super-ensemble”, we have computed segment-wize RMS end-to-end distances and the segment length deviation matrix (middle row in Figure 6). Because of the large size of the super-ensemble, these data are smoother. In general, they exhibit the same features as the initial ensemble obtained with all restraints. Unsurprisingly, the radius of gyration (23.7 Å) and width Γ = 48.2 Å, are unchanged.

While jack-knife resampling provides smooth estimates for segment-wise RMS end-to-end distances and the segment length deviation matrix derived from them, it does not directly provide an improved ensemble. However, during jack-knife resampling the modeling pipeline generates a large number of conformers that are consistent with at least *n*_*r*_-1 of *n*_*r*_ restraints. During ensemble refinement and reduction, the approach selects conformers that best fit *n*_*r*_-1 of *n*_*r*_ restraints. This provides a much improved basis set of conformers for fitting with all restraints. In our case, the 19 leave-one-restraint-out ensembles contain 1,119 conformers. Together with the 60 conformers in the initial ensemble, we have a basis that better samples conformational space and should thus allow for an improved fit. This expectation is indeed borne out, as can be seen by comparing the blue lines (fit with 1,179 pre-selected conformers) with the green lines (initial fit with 331 conformers) in Figure 5. In fact, the large raw ensemble may allow for some overfitting, i.e., the final ensemble may reproduce some features of the distance distributions that are uncertain, as can be seen by comparison with Figure 4. Overfitting of distance distributions is not easily quantified, as the distributions are solutions of an ill-posed problem. In future work, we will address this problem by considering fit quality of the primary data. In any case, the final ensemble fitted with the improved basis set provides smoother segment-wise RMS end-to-end distances and a smoother segment length deviation matrix than the initial ensemble. These characteristics are closer to the jack-knife mean estimates (bottom row in Figure 6). Therefore, we consider this ensemble with 129 conformers and mean overlap $\bar{o}=0.919$ as a better representation of the structure of the glycine-rich domain of hnRNP A1 than the initial fit. We note that jack-knife resampling generally provides a large number of conformers that are consistent with at least *n*_*r*_-1 of *n*_*r*_ distance distribution restraints. Since finding such conformers is computationally more expensive than ensemble reweighting, final computation of a “super-ensemble” is advantageous.

**FIGURE 7:**
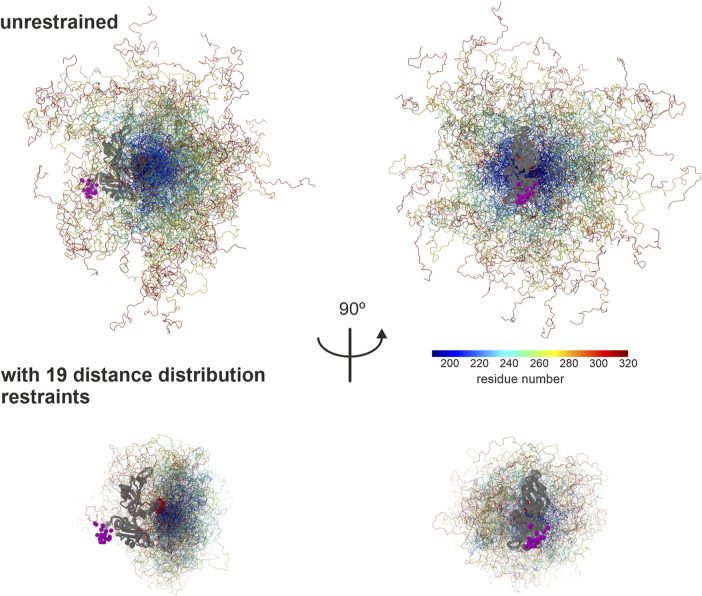
Ensemble model for the LCD of hnRNP A1. The two RRMs (PDB 2lyv, Barraud and Allain, 2013) are shown in gray, with the N terminus to the left in the left panel and to the front in the right panel. RRM 1 is on the bottom and RRM 2 on the top. Residues in the LCD are rainbow color coded from blue (residue 188) to red (320). Population of conformers is encoded by coil thickness, with the most populated conformer having a thickness of 0.25 Å. CA atoms of the N-terminus of RRM 1 are shown as purple spheres with radius 1.5 Å and CA atoms of the C-terminus of RRM 2 are shown as maroon spheres with the same radius. Visualization by ChimeraX (Goddard et al., 2018) via an MMMx script.

This final ensemble is visualized in Figure 7 together with an ensemble with the same number of 129 conformers randomly selected from an unrestrained raw ensemble with 2,146 conformers. For the unrestrained ensemble, we assumed uniform populations of the conformers. The restrained ensemble is much more compact. Residues 188–240 are located on the side of the two RRMs away from the N terminus, to a larger extent than in the unrestrained ensemble. Beyond residue 240, in the restrained ensemble the chain tends to backtrack toward the RRM, leaving the N-terminal side of the RRMs, but not the opposite side exposed. This exposure is also apparent in the unrestrained ensemble, where it is a purely geometrical effect. However, it is more pronounced in the restrained ensemble. Since the function of hnRNP A1 involves RNA binding, biological interpretation of this result requires further experiments including RNA binding, which are beyond the scope of this work and will be reported elsewhere. We may note here that, although a random-coil model obviously does not fit the weakly structured LCD of hnRNP A1 very well (Figure 6), it does predict scaling exponents ν ≈ 0.48, as they have earlier been observed for foldable proteins (Hofmann et al., 2012). Yet, the LCD is obviously not folded, as the distance distributions shown in Figures 4, 5 can be fitted only with a broad distribution of conformers.

## Discussion

### Model for the Unstructured Reference State and Detection of Weak Structure

For peptide chains of 100 or more residues, residue-specific Ramachandran statistics for loop regions is nicely consistent with Flory random coil scaling of segment RMS end-to-end distances (Figure 1B). Unfortunately, this approximation is not very good for short sections of 20–25 residues (Figure 3), which appear to be stiffer than predicted by a self-avoiding random walk of a freely jointed chain model, irrespective of the solvent conditions and condensation state. Attempts to extract random-coil parameters from DEER distance distributions are further confounded by convolution of the backbone end-to-end distance distribution with the rotamer distribution of the spin label side chain. Both problems are expected to lessen if the section length is increased. However, such a strategy shifts the mean of the distribution from the most favored range for DEER (25–40 Å) toward longer distances. This in turn shifts contributions by the longest conformers to a range where separation of the single-chain contribution from the intermolecular background becomes uncertain.

Therefore, our approach for recognizing deviation from an unstructured reference state is hybrid. First, we generate a large raw ensemble of 1,000–5,000 conformers that is in broad agreement with experimental restraints that are approximated by single Gaussian distributions in this step. The raw ensemble is based on residue-specific Ramachandran statistics for loop regions that is biased only by the experimental restraints. No residue-residue interaction potential is assumed. Second, we refine and contract this raw ensemble by fitting populations of conformers and by discarding conformers with very low population. In this step, we fit to full distance distributions that can have any shape. Third, we analyze scaling of segment-wise RMS end-to-end distances *R*_*k*_ with segment sequence length *k* and distribution of the *R*_*k*_ for given *k*. Broad distributions of the *R*_*k*_ and non-monotonic scaling of the mean *R*_*k*_ with *k* reveal deviations from random-coil behavior. These deviations can be heterogeneous along the sequence. We can map them to residue pairs by computing a segment length deviation matrix, whose elements are defined by Eq. 2. Indeed, based on our analysis of the full length hnRNP A1 construct, which contains both a strongly and a weakly ordered domain, we demonstrate that the segment analysis approach cannot only globally, but also locally uncover LCD structural deviations from random coil behavior.

This approach does not make any assumptions on the LCD beyond Ramachandran statistics. Even that assumption can, to some extent, be altered by the experimental restraints. With the numbers of conformers indicated above, it appears to be possible to fit experimental DEER distance distributions within their uncertainty for unstructured and weakly structured domains of 100–150 residues length on a current desktop computer. For longer domains or constructs, the number of required conformers in the raw ensemble may need to be reassessed or larger computational resources are required. It is also expected that the more structured a domain is, the more conformers need to be sampled in order to obtain a representative ensemble. In the limit of highly structured domains, the approach is not expected to be competitive with strategies that estimate variation from atomic-resolution structure.

### Robustness of Distance Distribution Restraints

The mapping of dipolar signals to distance distributions is not continuous in a mathematical sense of the term. Accordingly, computation of distance distributions corresponds to solving an ill-posed problem. The solution must be stabilized in some way, for instance, by regularization (Bowman et al., 2004; Jeschke et al., 2004; Chiang et al., 2005), by restricting it to a space of parametrized distribution functions (Stein et al., 2015), or by training a neural network for a restrained set of distance distributions (Worswick et al., 2018). Especially for weakly ordered or unstructured domains, fits by a single Gaussian function appear to work surprisingly well, as demonstrated here. In general, different analysis approaches provide distributions that may differ beyond their own uncertainty estimates (Figure 4). This indicates that model bias is a matter of concern. We find that differences between results from different approaches are minor if distributions are only moderately broadened and well within the preferred distance range of DEER. They can be large if the width is several tens of Ångström or if background separation becomes uncertain.

Ensemble fitting by distance distribution restraints is stabilized by the overlap criterion, as overlap of an ensemble-predicted distribution with the various distributions computed from the same experimental data varies much less than the shape of the various experiment-derived distributions. This finding applies to minor deviations between distance distributions computed by different approaches, as they are seen in Figure 4. If there exist major differences between distance distributions obtained by different approaches, data is of poor quality and should not be used for ensemble fitting. If in doubt, it may be prudent to perform ensemble modeling with distance distribution restraint sets derived by alternative approaches, as we have demonstrated here for the glycine-rich domain of hnRNP A1. In any case, we recommend to process experimental data sets by three different approaches: Tikhonov regularization, multi-Gaussian fitting, and neural network analysis. As seen in Figure 4, such comparison can reveal restraint uncertainty better than merely computation of uncertainty bands for a single data analysis approach. Comparison of the three distributions also reveals whether single-Gaussian restraints are a sensible choice for final ensemble fitting, as appears to be the case for hnRNP A1 188–320. This question is important, as fitting of a single Gaussian, on the one hand, is the most robust approach, but on the other hand, runs the largest risk of model bias.

For hnRNP A1, we have found that 19 distance distribution restraints suffice for stably characterizing substantial deviation of a 133-residue segment from random-coil behavior. Depending on how large such deviations are, the number of required distance distribution restraints may vary. Jack-knife resampling provides a general approach for testing whether a restraint set is sufficient for a given problem. If it is not, further restraints need to be added. For the case of hnRNP A1, we cannot exclude that less than 19 restraints would have sufficed. Establishing this would require a systematic analysis of how many and which restraints can be left out without substantially changing the proximity matrix. Such an analysis is computationally very expensive and beyond the scope of the current study.

### Resampling for Ensemble Model Validation

The number of distance distribution restraints *n*_res_ that enter into an ensemble model is expected to be in the range between 10 and 50. With much less than 10 restraints, it is unlikely that structure can be revealed except, perhaps, for very short peptides. Measuring much more than 50 restraints appears to be unrealistic because of the effort required in sample preparation. In this situation, jack-knife resampling appears to be a viable approach for ensemble validation, as we have demonstrated here for *n*_res_ = 19 on hnRNP A1 188–320. Jack-knife resampling requires *n*_res_ + 1 modeling runs, one of them with all restraints and *n*_res_ runs where one of the restraints is left out. We have shown that these modeling runs provide an improved basis set of conformers that can be used as input for a final ensemble fit. Regarding computational effort, such jack-knife resampling is certainly feasible for domains with up to 150 residues and for up to 50 restraints on current desktop computers. Further automation in software packages is required to make jack-knife resampling convenient. This will be pursued in further development of MMMx.

### Choice of Labeling Sites

For characterizing the LCD of hnRNP A1, we relied on three reference sites in the structured domain of the protein. Such a choice is advantageous in cases where a structured domain exists and where the localization of the intrinsically disordered domain with respect to the structured domain is of interest. Could we have characterized weak order of the LCD on its own, provided that it would have been the same as in the presence of the RRM? To answer this question, we performed a computational experiment that assumed the final ensemble of our study as ground truth. In addition to the three experimental intra-LCD restraints (231–271, 231–316, and 271–316) we simulated distance distributions for 16 additional intra-LCD site pairs. These pairs were selected from LCD sites that we had also labeled in our experiments (190, 197, 223, 231, 252, 271, 297, and 316) by limiting sequence distance to no more than 85 residues, i.e., the maximum sequence separation within the LCD in our experimental restraints (Supplementary Table S1). With these restraints, we generated a raw ensemble of 1,290 conformers and then performed ensemble reweighting with the EnsembleFit module of MMMx. The final ensemble fits the 16 simulated and three experimental restraints with an overlap deficiency of 0.047. As seen in Supplementary Figure S6, site-resolved compaction of the LCD is reasonably well reproduced with this set of restraints, with notable deviations near the C terminus. In our original restraint set, residue 316 is localized with respect to the RRM by three restraints, whereas in the simulated restraint set, due to our chosen maximum cutoff segment length of 85 residues, it is not localized with respect to any site upstream of residue 231. This suggests that some site pairs with long sequence separation must be included in the set in order to avoid that structural features are missed.

## Conclusion

Ensemble modeling and refinement with label-to-label distance distributions measured by EPR pulsed dipolar spectroscopy is a feasible approach for characterizing weak structure in protein domains. Our approach involves generation of a basis set of conformers that is consistent with Ramachandran statistics for loop residues and with restraints. This raw ensemble is refined and contracted by fitting to distance distributions restraints. It is then analyzed in terms of segment-wize RMS end-to-end distributions. For estimating uncertainty and validating restraints, we recommend computation of distance distributions from primary data by several alternative approaches and jack-knife resampling of the restraints in ensemble modeling. The current implementation is viable up to about 150 residues and up to about 50 distance distribution restraints on current desktop computers. Extension of these limits appears to be feasible. Our approach could be extended to integrative modeling that uses restraints from further experimental techniques in ensemble refinement.

## Data Availability

The raw data supporting the conclusions of this article will be made available by the authors, without undue reservation.

## Footnotes

1
http://www.biochemistry.ucla.edu/Faculty/Hubbell/.

2
http://www.github.com/gjeschke/MMMx.

3
http://www.biochemistry.ucla.edu/Faculty/Hubbell/.
