# Supplementary material for: Characterization of Weak Protein Domain Structure by Spin-Label Distance Distributions
Source: Front Mol Biosci. 2021 Apr 12;8:636599. doi: 10.3389/fmolb.2021.636599 (PMC8072059; doi:10.3389/fmolb.2021.636599)
Supplement: Supplementary file 1 [file datasheet1.pdf]

Supplementary Material

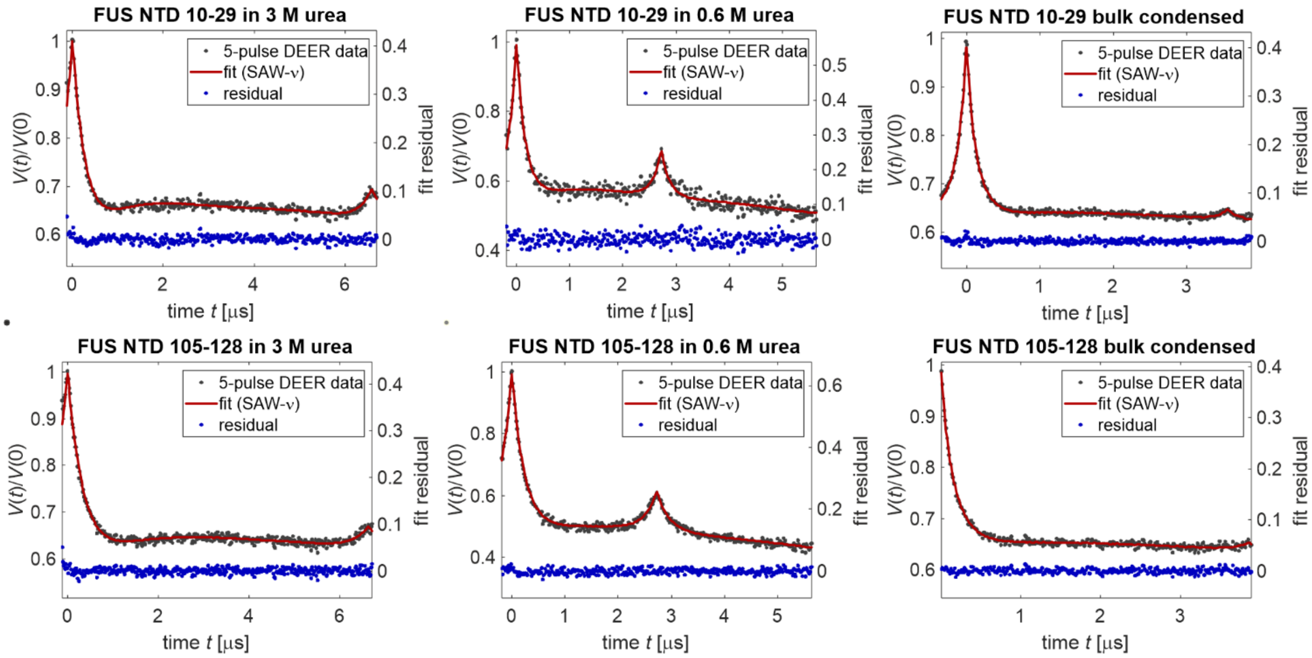

Figure S1 Fit of primary DEER data of FUS NTD (black dots) in DeerLab by an SAW-ν distribution (red, left ordinate) fitted simultaneously with a monoexponential background function. The fit residual is shown as blue (right ordinate). Signals from minor pathways at the end of 4-pulse DEER data or at the middle of 5-pulse DEER data were included in the kernel (Fabregas *et al.*, 2020).

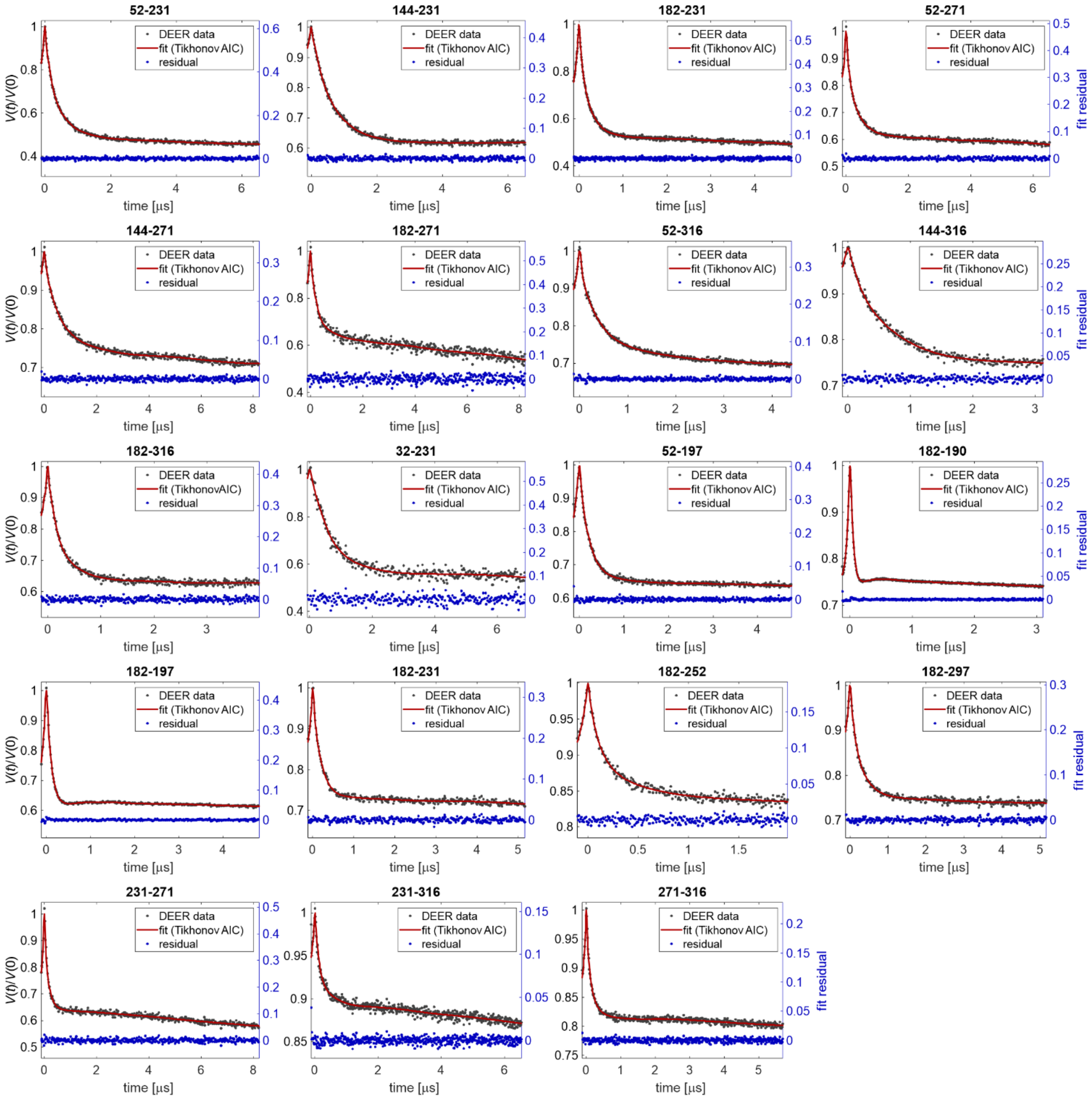

Figure S2 Fit of primary DEER data of hnRNP A1 LCD (black dots, left ordinate) in DeerLab by Tikhonov regularization with the regularization parameter α selected by AIC (red) and the fit residual (blue dots, right ordinate).

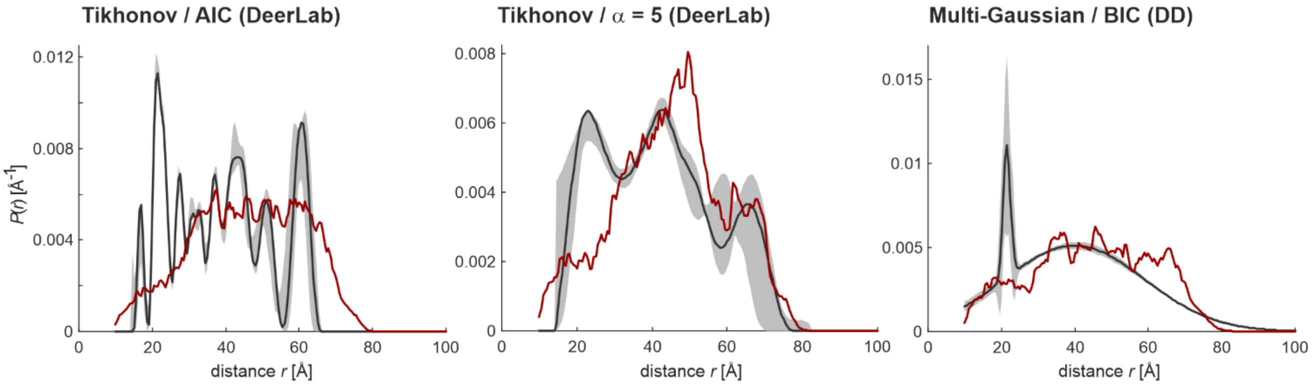

Figure S3 Effect of undersmoothing on distance distribution overlap. Experimental distributions are shown as black lines with grey uncertainty bands and ensemble fits as red lines. For the DEER data set of site pair 52-316 in hnRNP A1, an unrealistically small regularization parameter α = 0.14 is suggested by the Akaike information criterion (AIC), which leads to splitting of the distance distribution into several narrow peaks and consequently mediocre overlap of 0.731 (left). With fixed regularization parameter α = 5, the overlap improves to 0.835 (middle). In contrast, a single unrealistically narrow Gaussian component in a multi-Gaussian fit is less detrimental, allowing for an overlap of 0.846 (right).

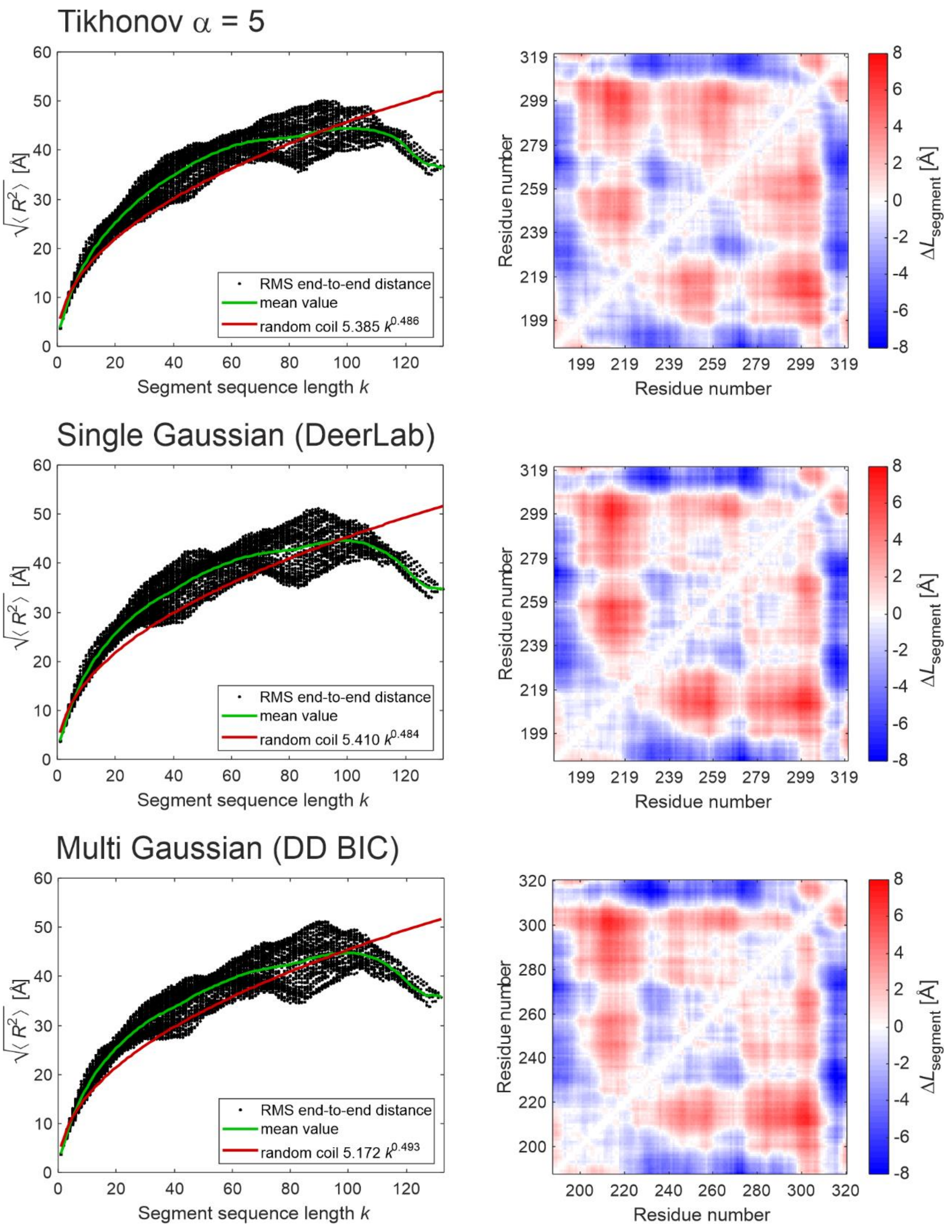

Figure S4 Ensemble analysis for the LCD of hnRNP A1 with different restraint sets. The left column shows RMS CA-CA distances for all segments of the LCD (residues 188-320) as a function of segment sequence length (black dots) and their mean values per segment sequence length (green line). The right column shows the deviation of the RMS CA-CA distances from the mean value for this segment sequence length (green line in the left column). Red hues correspond to segments more extended than the average and blue hues to segments more compact than the average.

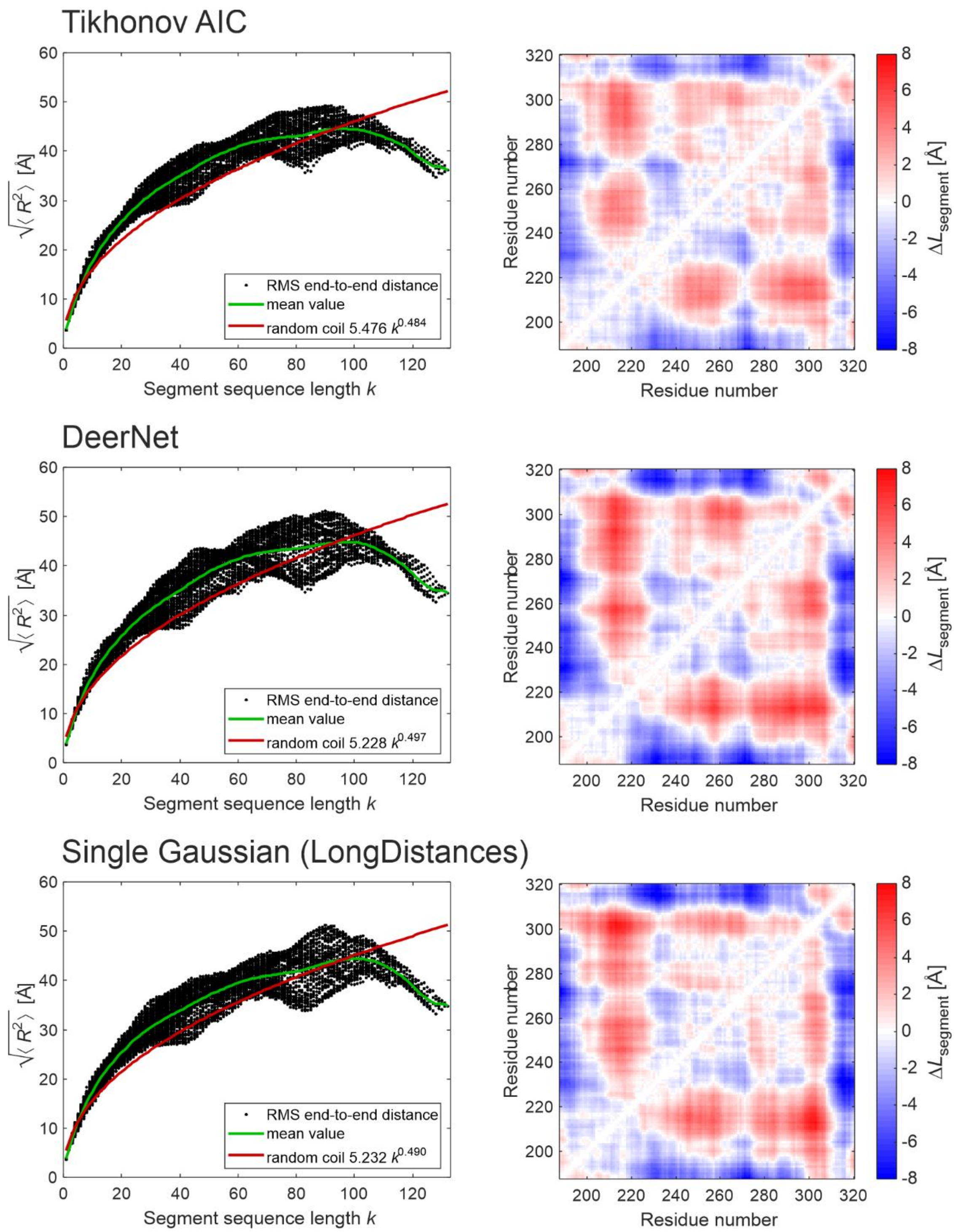

Figure S5 Ensemble analysis for the LCD of hnRNP A1 with different restraint sets. The left column shows RMS CA-CA distances for all segments of the LCD (residues 188-320) as a function of segment sequence length (black dots) and their mean values per segment sequence length (green line). The right column shows the deviation of the RMS CA-CA distances from the mean value for this segment sequence length (green line in the left column). Red hues correspond to segments more extended than the average and blue hues to segments more compact than the average.

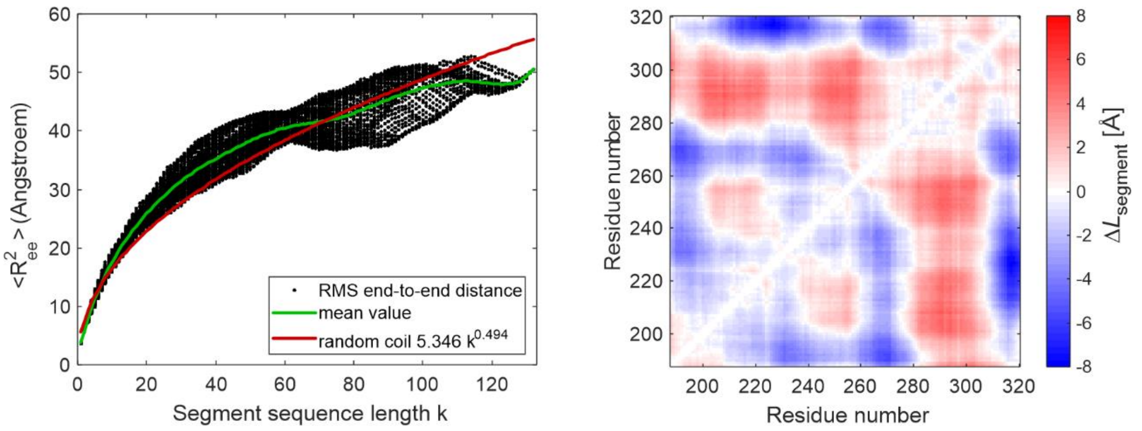

Figure S6 Ensemble analysis after refit of the final ensemble using only intra-LCD restraints. The three experimental restraints for site pairs 231-271, 231-316, 271-316 were combined with 16 distance distribution restraints simulated from the final ensemble for the site pairs listed in Table S1. The left panel shows RMS CA-CA distances for all segments of the LCD (residues 188-320) as a function of segment sequence length (black dots) and their mean values per segment sequence length (green line). The right panel shows the deviation of the RMS CA-CA distances from the mean value for this segment sequence length (green line in the left column). Red hues correspond to segments more extended than the average and blue hues to segments more compact than the average.

Table S1 Simulated intra-LCD restraints for refitting of the final ensemble

**Site1**	**Site2**	**$\langle r \rangle_{sim}$ [Å]**	**σ_{sim} [Å]**
190	197	19.8	5.6
190	223	30.1	11.4
190	231	30.2	11.3
190	252	38.1	11.3
190	271	33.1	12.0
197	223	28.4	10.1
197	231	28.9	11.1
197	252	39.1	12.3
197	271	35.6	13.3
223	231	20.1	5.5
223	252	32.5	10.5
223	271	35.3	11.7
223	297	43.2	14.0
252	271	28.1	9.6
252	297	40.2	12.4
252	316	38.1	13.9
